# Hybrid Manta Ray Foraging Algorithm with Cuckoo Search for Global Optimization and Three-Dimensional Wireless Sensor Network Deployment Problem

**DOI:** 10.3390/biomimetics8050411

**Published:** 2023-09-05

**Authors:** Meiyan Wang, Qifang Luo, Yuanfei Wei, Yongquan Zhou

**Affiliations:** 1 College of Artificial Intelligence, Guangxi Minzu University, Nanning 530006, China; 202120812001895@stu.gxmzu.edu.cn (M.W.);; 2 Guangxi Key Laboratories of Hybrid Computation and IC Design Analysis, Nanning 530006, China; 3 Faculty of Information Science and Technology, Universiti Kebangsaan Malaysia, Bangi 43600, Selangor, Malaysia; 4 Xiangsihu College, Guangxi Minzu University, Nanning 530225, China

**Keywords:** manta ray foraging optimization, cuckoo search, AMRFOCS, benchmark function, three-dimensional WSN, metaheuristic algorithm

## Abstract

In this paper, a new hybrid Manta Ray Foraging Optimization (MRFO) with Cuckoo Search (CS) algorithm (AMRFOCS) is proposed. Firstly, quantum bit Bloch spherical coordinate coding is used for the initialization of the population, which improves the diversity of the expansion of the traversal ability of the search space. Secondly, the dynamic disturbance factor is introduced to balance the exploratory and exploitative search ability of the algorithm. Finally, the unique nesting strategy of the cuckoo and Levy flight is introduced to enhance the search ability. AMRFOCS is tested on CEC2017 and CEC2020 benchmark functions, which is also compared and tested by using different dimensions and other state-of-the-art metaheuristic algorithms. Experimental results reveal that the AMRFOCS algorithm has a superior convergence rate and optimization precision. At the same time, the nonparametric Wilcoxon signed-rank test and Friedman test show that the AMRFOCS has good stability and superiority. In addition, the proposed AMRFOCS is applied to the three-dimensional WSN coverage problem. Compared with the other four 3D deployment methods optimized by metaheuristic algorithms, the AMRFOCS effectively reduces the redundancy of sensor nodes, possesses a faster convergence speed and higher coverage and then provides a more effective and practical deployment scheme.

## 1. Introduction

A wireless sensor network (WSN) is a wireless network composed of a set of sensor nodes, which is applied to monitor and record all aspects of the region of interest [1]. WSNs have been widely used in disaster relief, public safety, smart-city construction [2], agricultural monitoring and other fields [3,4]. One of the important problems in WSNs is optimizing the deployment of sensor nodes, which determines the regional coverage, overall network connectivity and network service quality of the WSN [5].

At present, the deployment of WSNs on a two-dimensional plane has been relatively well studied, while three-dimensional deployment has been less researched. The 3D deployment of wireless sensor networks is divided into two types: one is deployed in the 3D space, and the other is deployed on the surface [6]. The coverage optimization of wireless sensor networks on 3D surfaces has become a heated topic in wireless sensor network research due to its complexity and practicality [7].

Recently, researchers have applied a variety of bionic metaheuristic swarm intelligence algorithms to improve the performances of wireless sensor networks, including the extended coverage area, network lifetime, routing protocol, sensor random deployment, energy consumption model, etc. Table 1 specifically demonstrates the optimization performance of swarm intelligence algorithms in wireless sensor networks in recent years.

The swarm intelligence (SI) category has received abundant attention recently [8]. SI is a group-based algorithm driven by biological group behavior [9]. The diverse species in nature exhibit unique behaviors and habits. Swarm intelligence algorithms are mainly based on the foraging, communication and reproductive behaviors of biological organisms. Representative examples of these behaviors include Particle Swarm Optimization (PSO) [10], Ant Colony Optimization (ACO) [11], Cuckoo Search (CS) [12] algorithms, Artificial Bee Colony (ABC) [13], the Grey Wolf Optimizer (GWO) [14], the Butterfly Optimization Algorithm (BOA) [15], the Whale Optimization Algorithm (WOA) [16], Harris hawks optimization (HHO) [17], Manta Ray Foraging Optimization (MRFO) [18], Jellyfish Search (JS) [19], the Honey Badger Algorithm (HBA) [20], Sand Cat Swarm Optimization (SCSO) [21], the Dandelion Optimizer (DO) [22], the Coati Optimization Algorithm (COA) [23], the Fire Hawk Optimizer (FHO) [24] and the Sea-horse Optimizer (SHO) [25].

MRFO is a swarm intelligence algorithm proposed in 2020. Its inspiration comes from the foraging behavior of manta rays, which simulates three different foraging strategies: chain foraging, spiral foraging and flip-bucket foraging. In the foraging process, the conversion of different foraging methods is formulated as a conversion based on global and local optimization. MRFO has an excellent global optimization ability, as it has fewer adjustable parameters, simpler implementations and lower computational cost. It has been widely used in electrical engineering [26], civil engineering [27], image segmentation [28], photovoltaic models [29], network engineering [30] and structural design [31].

Although MRFO has been applied in many fields, previous studies have shown that its exploration ability is weak [32], and it is easy to stagnate the local optimum [33]. In order to solve this problem, this paper improves MRFO and applies it to WSN node deployment on a three-dimensional surface.

The structure of this paper is presented as follows: Section 2 focuses on the basic algorithm. In Section 3, the approach is proposed. Section 4 describes the experimental results and comparison of the CEC2017 and CEC2020 benchmark functions with AMRFOCS. Section 5 is about the application of AMRFOCS in the development optimization of wireless sensor nodes, and Section 6 contains conclusions and future work.

**Table 1:** The differences in the applications of different algorithms in WSNs.

Algorithm	Application in WSNs	Key Features	Advantages	Limitations
PSO [2]	Coverage maximization, network lifetime	Swarm-based optimization, global search capability	Fast convergence, reduce costs, coverage enhancement	Scale limitations, obstacles not considered
CS [3]	Node localization	The flight characteristics of the cuckoo	Better convergence, calculation accuracy	Time consuming, complex implementation
WOA [34]	Coverage optimization	Social Behavior of humpback whales	High coverage, low deployment cost	2D, convergence speed
GWO [35]	Coverage optimization	Group hunting behavior of gray wolves	Easy implementation, high search efficiency	Time consuming, 2D
SSA [36]	Network data aggregation	Squirrel foraging behavior	Low energy consumption, high accuracy	2D, convergence speed
SMA [37]	Node localization, 3D	Behavior of slime mold	Low complexity, high convergence	CPU time, high memory
ABC [38]	Routing protocol	Honey bee behavior	Reduced convergence delay, low energy consumption	Time consuming, complex implementation
HBA [39]	Smart city	Foraging behavior of honey badgers	Low energy consumption, high accuracy	Scale limitations, complex implementation
BOA [40]	Energy efficiency	Behavior of butterflies	Low complexity, high efficiency	Complex implementation
ACO [40]	Energy efficiency	Foraging behavior of ants	Low complexity	High memory
PIO [41]	Coverage optimization	Pigeon homing behavior	Better convergence, high efficiency	2D, time consuming

## 2. Preliminary

### 2.1. Manta Ray Foraging Optimization

The Manta Ray Foraging Optimization (MRFO) algorithm is a new population-based swarm intelligence optimization algorithm used to simulate animal foraging behaviors and characteristics. The main inspiration of this algorithm is the special and manifold foraging behavior of manta rays. In order to distinguish their different behaviors, they are described as chain foraging, spiral foraging and flipping foraging. In particular, if the physical space of the manta ray motion corresponds to the search space of the MRFO algorithm, the position $x_{i}^{d}(t)$ of the manta ray is the solution in the searching region, and the place $x_{best}^{d}(t)$ of the optimal food source is the optimal solution in the search region.

During the cyclone foraging stage, the manta ray swims forward spirally. According to the value of the convergence factor (*C*) (the ratio of the number of iterations to the maximum number of iterations), it is decided to choose the best position obtained at present, which is helpful for development or to choose the reference random position, which is helpful for exploration:

(1) $$\beta =2e^{r_{1}\frac{T_{Max}-t+1}{T_{Max}}}\cdot \sin(2\pi \cdot r_{1})$$

(2) $$x_{i}^{d}(t+1)=\left\{ \begin{array}{l} x_{best}^{d}+r\cdot [x_{best}^{d}(t)-x_{i}^{d}(t)]+\beta \cdot [x_{best}^{d}(t)-x_{i}^{d}(t)]\;\;i=1\\ x_{best}^{d}+r\cdot [x_{i-1}^{d}(t)-x_{i}^{d}(t)]+\beta \cdot [x_{best}^{d}(t)-x_{i}^{d}(t)]\;\;i=2,\ldots ,N \end{array}\right.$$

where β is the weight coefficient, $T_{Max}$ is the maximum number of iterations and $r_{1}$ is a random number in [0, 1]:

(3) $$x_{\mathrm{rand}}^{d}=r\cdot (Up^{d}-Low^{d})+Low^{d}$$

(4) $$x_{i}^{d}(t+1)=\left\{ \begin{array}{l} x_{rand}^{d}+r\cdot [x_{rand}^{d}(t)-x_{i}^{d}(t)]+\beta \cdot [x_{rand}^{d}(t)-x_{i}^{d}(t)]\;\;i=1\\ x_{rand}^{d}+r\cdot [x_{i-1}^{d}(t)-x_{i}^{d}(t)]+\beta \cdot [x_{rand}^{d}(t)-x_{i}^{d}(t)]\;\;i=2,\ldots ,N \end{array}\right.$$

where $x_{rand}^{d}$ denotes an arbitrary position in the searching region and $Up^{d}$ and $Low^{d}$ are the upper and lower limits of the *d*th dimension, respectively.

In the chain foraging stage, the position of the manta ray individual is updated according to the previous individual position and the optimal food source position:

(5) $$\alpha =2\cdot r\cdot \sqrt{\left|\log(r)\right|}$$

(6) $$x_{i}^{d}(t+1)=\left\{ \begin{array}{l} x_{i}^{d}+r\cdot [x_{best}^{d}(t)-x_{i}^{d}(t)]+\alpha \cdot [x_{best}^{d}(t)-x_{i}^{d}(t)]\;\;\;i=1\\ x_{i}^{d}+r\cdot [x_{i-1}^{d}(t)-x_{i}^{d}(t)]+\alpha \cdot [x_{best}^{d}(t)-x_{i}^{d}(t)]\;\;\;i=2,\ldots ,N \end{array}\right.$$

where $x_{i}^{d}(t)$ presents the position of the *i*th individual at time *t* in the *d*th dimension, *r* is a random vector within [0, 1] and *a* is a weight coefficient. The position update of the *i*th individual is determined by the position $x_{i-1}^{d}(t)$ of the (*i* − 1)th current individual and the position $x_{best}^{}(t)$ of the food.

In somersault foraging, the new positions can be represented as

(7) $$x_{i}^{d}(t+1)=x_{i}^{d}(t)+S\cdot r\cdot \left(x_{best}^{d}(t)-x_{i}^{d}(t)\right),\;i=1,\ldots \ldots ,N$$

where *S* = 2 and *S* is the somersault factor, which determines the search range of each flip.

### 2.2. Cuckoo Search Algorithm

The main inspiration for the Cuckoo Search (CS) algorithm comes from the interesting parasitic brooding behavior of cuckoos. In order to describe the Cuckoo Search algorithm, the following three idealized rules are used: First, each cuckoo lays an egg in a nest at a time, and the nest is randomly selected. Second, the nest with high-quality eggs is retained as a continuous generation; that is, the optimal solution is retained. Third, it is assumed that the existing host nest is invariable, and the host discovers the eggs laid by the cuckoo with a probability of 0 ≤ *P* ≤ 1 and then throws away the eggs or discards the existing nest.

The parasitic nest of cuckoo eggs represents the solution of the search space, and the location of the parasitic nest represents the fitness value of the solution. In the optimization process of CS, parameter *P* affects the transition between local development and global search. The nest location update mechanism conforms to the *Levy* flight, and the equation is updated as follows:

(8) $$x_{i}^{t+1}=x_{i}^{t}+\alpha ⊗Levy(\lambda )$$

where $x_{i}^{t}$ represents the position of the *i*th bird nest in the *k*th generation nest, ⊗ represents point-to-point multiplication, *a* represents the step length control quantity and Levy(λ) is a random search path.

## 3. The Proposed Method

In MRFO, the transition of the different foraging modes of manta rays is only determined by the comparison of random numbers. The imbalance between exploration and exploitation greatly affects the performance of the algorithm. In order to enhance the performance of MRFO, this paper improves it from two aspects: First, the dynamic disturbance factor strategy is introduced to balance exploration and development to ensure accuracy, and the AMRFO algorithm is proposed. Secondly, the CS algorithm is mixed on the basis of AMRFO, and the exploration ability of CS is used to enhance the global convergence ability of the algorithm and to avoid low efficiency and the local optimum.

### 3.1. Dynamic Perturbation Factor Strategy

In the original MRFO algorithm, the excess of the exploitation and exploration of cyclone foraging is determined by the comparison between the random number (rand) and the convergence factor (*C*) (the ratio of the number of iterations to the maximum number of iterations). When the random number is less than the convergence factor, the manta ray carries out the global search and expands the exploration range. When the random number is greater than the convergence factor, the manta ray performs a local search to improve the efficiency of the local search. However, the disadvantage is that the early and late decline in the convergence factor is the same, resulting in the inability to perform a local search better in the early stage and the inability to perform a partial search more effectively in the later step, which will lead to disadvantages in handling actual optimization problems. In most cases, it cannot be guaranteed that the global optimal solution can be obtained at the end of the convergence; there will be premature convergence, and the model will fall into a local optimum later. The convergence factor is generally improved from linear to nonlinear so that the previous convergence factor can be smoothly decreased and the global exploration capability can be augmented. The steep decline at the later stage enhances its local mining capacity. In this paper, a new dynamic factor strategy will be introduced to improve its accuracy. The perturbation factor *P* is shown in Equation (9), and the updated parameter *M* is shown in Equation (10):

(9) $$P=randn\cdot \left(\sin^{\omega }(\frac{\pi }{2}\cdot \frac{t}{t_{\max}})+\cos(\frac{\pi }{2}\cdot \frac{t}{t_{\max}})-1\right)$$

**Figure 1:**
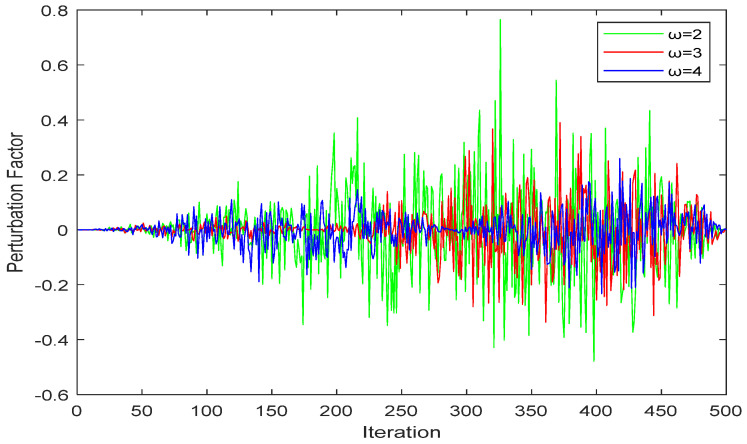
Variation in *M* value with different values of parameter ω.

(10) M=a⋅(2×rand−1)+P

where *randn* represents a random number that obeys a Gaussian normal distribution; ω represents a constant that determines the peak position of the perturbation factor; and *M* is a part of the position update, which balances the global search and local exploitation in cyclone foraging. Figure 1 shows that *M* changes with the increase in the number of iterations. It can be embraced from the comparison in the figure that when ω=3, the amplitude of the disturbance factor is large and stable, and it is also verified in subsequent experiments that it can improve the performance of the algorithm.

### 3.2. Hybrid CS with AMRFO

All population-based metaheuristic algorithms such as MRFO and CS essentially solve optimization problems by maintaining a compromise between development and exploration. In MRFO, chain foraging and flip-bucket foraging update the position of the search agent around the optimal position, focusing on the development performance of the algorithm. However, the special features of spiral foraging are used to improve global exploration. CS has a special flight strategy that cannot easily fall into the local optimum and has a strong global search ability. CS is embedded into AMRFO to further optimize the algorithm’s performance, prevent the model from falling into the local optimum and improve the convergence rate and preciseness. 

### 3.3. The Proposed AMRFOCS

**Figure 2:**
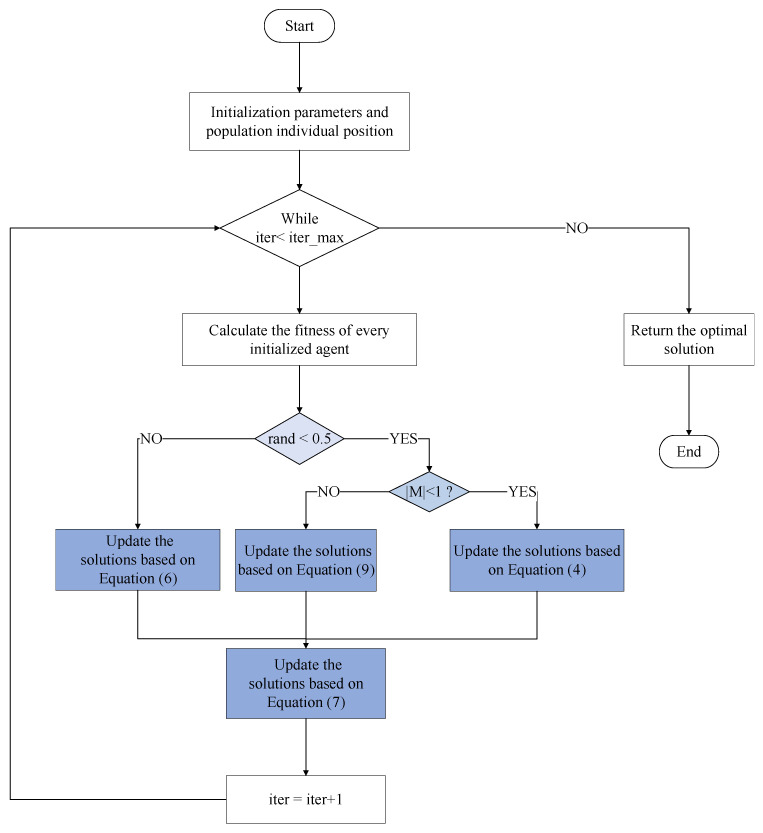
Flowchart of proposed AMRFOCS method.

To express the proposed algorithm more clearly, Algorithm 1 gives the pseudo code of the proposed AMRFOCS, and Figure 2 is the workflow chart of the proposed AMRFOCS algorithm.
**Algorithm 1:** AMRFOCS1. Input: The number of generations (*T*), size of the population (*N*), and the upper and lower bounds Up and Low.2. Output: Optimal solution *xbest*.3. Initialize the population and parameters α,β,S,P,M,ω4. Compute the fitness of every initialized agent and sort all agents according to their fitness values.5. while *t* < *T* do:6.  if rand < 0.5 then7.    if *|M|* < 1 then8.      Update $x_{i}$ based on Equation (4).9.    else if then10.      Update $x_{i}$ on the usage of Equation (9).11.    end if12.  else if then13.     Update $x_{i}$ on the usage of Equation (6)14.  end if15.  for *i* = 1:*N* do16.     Update $x_{i}$ based on Equation (7).17.     if $f(x_{i}(t+1))\;<\;f(x_{\mathrm{best}})$ then18.       $x_{best}=x_{i}(t+1)$19.     end if20.   end for21.   Sort the new population according to fitness.22.   t = t+123. end while24. return 

### 3.4. The Computational Complexity of AMRFOCS

One of the main metrics of the optimization algorithm is the execution time. The modifications of AMRFOCS include the dynamic perturbation factor and the hybridizing of CS. When the population size, the dimension of the optimization problem and the maximum number of iterations are set to *N*, *D* and *T*, respectively, the complexity of AMRFOCS can be calculated in the following ways: The complexity of the initialization phase is O(N×D), the complexity of the dynamic disturbance factor is O(N) and the complexity of the algorithm update solution is O(N×D). When performing T iterations, the total time complexity of AMRFOCS is O(N×D+T×(N+N×D+N×D+N×D))=O(N×(D+T+3×T×D)), which is higher than the O(T×N×D) of the original MRFO.

## 4. Experimental Results and Discussion

In this section, in order to evaluate the performance of the proposed AMRFOCS and verify its effectiveness, two highly respected function families are used: thirty CEC 2017 and ten CEC 2020 benchmark functions. The improved AMRFOCS is compared with other well-known optimization algorithms in multiple dimensions. Information about the benchmark function is shown in Table 2 and Table 3. The environmental conditions of all the simulation experiments involve Intel^®^ Core™ i7-9700 CPU @ 3.00 GHz, 16 GB RAM, the Windows 10 operating system and the MATLAB 2021b platform.

### 4.1. Comparison of AMRFOCS with Other Algorithms on CEC2017

#### 4.1.1. Analysis and Discussion of Results 

In this section, AMRFOCS is compared with eight other metaheuristic algorithms, including ACO [42], ABC [43], SMA [44], PSO [45], GA, DE [46], WOA [46] and MRFO [18]. In order to ensure the fairness and rigor of the experiment, parameters are set in each selected algorithm, as shown in Table 4. Except for the parameters in Table 4, the other parameters are consistent. Each algorithm was run independently 30 times with dimensions of 30 and 50 and a population size of 50 and 30, and the maximum number of iterations was set to 500. The results of 30 independent runs are listed in Table 5 and Table 6.

Table 5 and Table 6 show four indicators, namely the mean value (mean), standard deviation (std), minimum value (min) and maximum value (max), as well as the sort value of the algorithm. In addition, at the 5% significance level, the Wilcoxon rank-sum test [47] is used to affirm whether AMRFOCS has a significant contribution to other algorithms. “-” represents ‘‘not applicable’’, which means that the best algorithm cannot be statistically compared with itself in the rank-sum test [39]. Table 5 and Table 6 give the algorithm’s ranking in different test functions and the *p*-value of the rank-sum test. The table’s bold data are the eight algorithms’ optimal minimum values (maximum values, mean values or standard deviation). Additionally, the last row of Table 5 and Table 6 lists three symbols (+/−/=) to show the number of functions whereby AMRFOCS has a superior (+) performance, the number of functions whereby AMRFOCS has the same behavior as other algorithms (=) and the number of functions whereby AMRFOCS is at a disadvantage.

Table 5 and Table 6 show that AMROCS provides topnotch results in most functions, especially when dealing with unimodal functions (F1, F3), multimodal (F5), mixed (F11, F12, F13, F16, F19, F20 and F17) and combined functions (F20, F21, F24 and F28–F30). Therefore, AMRFOCS has the smallest average rank ranking, with more than 32% of the features in these tests achieving an optimal performance. This experimental result confirms the effective exploration and utilization ability of the proposed AMRFOCS in exploring and accurately utilizing the optimal solution and avoiding many local optimums in high-dimensional combinatorial functions. The table also shows the *p* value of AMRFOCS and the other algorithms, which confirms the significant difference between the AMRFOCS proposed in this paper and other algorithms. Therefore, AMRFOCS is a faithful and steady-going algorithm.

**Table 2:** CEC2017 benchmark functions summary [48].

Type	No.	Functions	Range	$F_{\min}$
Unimodal Function	F1	Shifted and Rotated Bent Cigar Function	[−100, 100]	100
	F2	Shifted and Rotated Sum of Different Power Function	[−100, 100]	200
	F3	Shifted and Rotated Zakharov Function	[−100, 100]	300
Simple Multimodal Functions	F4	Shifted and Rotated Rosenbrock’s Function	[−100, 100]	400
	F5	Shifted and Rotated Rastrigin’s Function	[−100, 100]	500
	F6	Shifted and Rotated Expanded Scaffer’s F6 Function	[−100, 100]	600
	F7	Shifted and Rotated Lunacek Bi_Rastrigin Function	[−100, 100]	700
	F8	Shifted and Rotated Noncontinuous Rastrigin’s Function	[−100, 100]	800
	F9	Shifted and Rotated Levy Function	[−100, 100]	900
	F10	Shifted and Rotated Schwefel’s Function	[−100, 100]	1000
Hybrid Functions	F11	Hybrid Function 1 (N = 3)	[−100, 100]	1100
	F12	Hybrid Function 2 (N = 3)	[−100, 100]	1200
	F13	Hybrid Function 3 (N =3)	[−100, 100]	1300
	F14	Hybrid Function 4 (N = 4)	[−100, 100]	1400
	F15	Hybrid Function 5 (N = 4)	[−100, 100]	1500
	F16	Hybrid Function 6 (N = 4)	[−100, 100]	1600
	F17	Hybrid Function 6 (N =5)	[−100, 100]	1700
	F18	Hybrid Function 6 (N =5)	[−100, 100]	1800
	F19	Hybrid Function 6 (N =5)	[−100, 100]	1900
	F20	Hybrid Function 6 (N = 6)	[−100, 100]	2000
Composition Functions	F21	Composition Function 1 (N = 3)	[−100, 100]	2100
	F22	Composition Function 2 (N = 3)	[−100, 100]	2200
	F23	Composition Function 3 (N = 4)	[−100, 100]	2300
	F24	Composition Function 4 (N = 4)	[−100, 100]	2400
	F25	Composition Function 5 (N = 5)	[−100, 100]	2500
	F26	Composition Function 6 (N = 5)	[−100, 100]	2600
	F27	Composition Function 7 (N = 6)	[−100, 100]	2700
	F28	Composition Function 8 (N = 6)	[−100, 100]	2800
	F29	Composition Function 9 (N = 3)	[−100, 100]	2900
	F30	Composition Function 10 (N = 3)	[−100, 100]	3000

**Table 3:** The CEC2020 benchmark functions [49].

Type	No.	Functions	Range	$F_{\min}$
Unimodal Function	F1	Shifted and Rotated Bent Cigar Function (CEC 2017 F1)	[−100, 100]	100
Basic Functions	F2	Shifted and Rotated Schwefel’s Function (CEC 2014 F11)	[−100, 100]	1100
	F3	Shifted and Rotated Lunacek Bi_Rastrigin Function (CEC 2017 F7)	[−100, 100]	700
	F4	Expanded Rosenbrock’s plus Griewangk’s Function (CEC2017 F19)	[−100, 100]	1900
Hybrid Functions	F5	Hybrid Function 1 (N = 3) (CEC 2014 F17)	[−100, 100]	1700
	F6	Hybrid Function 2 (N = 4) (CEC 2017 F16)	[−100, 100]	1600
	F7	Hybrid Function 3 (N = 5) (CEC 2014 F21)	[−100, 100]	2100
Composition Functions	F8	Composition Function 1 (N = 3) (CEC 2017 F22)	[−100, 100]	2200
	F9	Composition Function 2 (N = 4) (CEC 2017 F24)	[−100, 100]	2400
	F10	Composition Function 3 (N = 5) (CEC 2017 F25)	[−100, 100]	2500

**Table 4:** Parameter settings of different algorithms.

Algorithm	Parameters	Setting Value
ABC	*a*	1
	*k*	[1, 10]
	*p*	[−1, 1]
ACO	*r*	0.9
	*P*	0.2
	*y*	[−5, 5]
	*s*	0.1
DE	*f*	0.5
	*c*	0.9
GA	*l*	20
	*g*	0.9
	*c*	1
	*s*	0
SMA	*z*	0.03
PSO	*a*	2.0
	*c*	2.0
WOA	*b*	1
MRFO	*S*	2

**Table 5:** Comparison of results obtained—CEC2017 benchmark functions (30D).

Function		ABC [43]	ACO [42]	DE [46]	GA	SMA [44]	PSO [45]	WOA [46]	MRFO	AMRFOCS
F1	Min	1.0154 × 10^8^	9.7148 × 10^10^	4.1095 × 10^6^	4.8990 × 10^10^	1.5430 × 10^3^	1.2997 × 10^7^	5.9003 × 10^8^	1.4427 × 10^2^	3.1760 × 10^2^
	Max	8.3189 × 10^8^	1.5513 × 10^11^	1.9979 × 10^7^	1.0217 × 10^11^	2.2200 × 10^4^	2.5143 × 10^7^	5.9382 × 10^9^	2.0702 × 10^4^	1.9893 × 10^4^
	Mean	3.1937 × 10^8^	1.2948 × 10^11^	1.1488 × 10^7^	8.2198 × 10^10^	7.1400 × 10^3^	1.9757 × 10^7^	2.1909 × 10^9^	5.1159 × 10^3^	4.2103 × 10^3^
	Std	1.7466 × 10^8^	1.3586 × 10^10^	4.4221 × 10^6^	1.2920 × 10^10^	5.7760 × 10^3^	2.6789 × 10^6^	1.2084 × 10^9^	6.2854 × 10^3^	4.4114 × 10^3^
	Rank	6	9	4	8	3	5	7	2	1
	*p*-value	3.0199 × 10^−11^	3.0199 × 10^−11^	3.0199 × 10^−11^	3.0199 × 10^−11^	1.4643 × 10^−10^	3.0199 × 10^−11^	3.0199 × 10^−11^	3.0199 × 10^−11^	-
F2	Min	1.3826 × 10^39^	3.0174 × 10^42^	3.7900 × 10^20^	2.6307 × 10^39^	3.9166 × 10^8^	6.9869 × 10^5^	1.8526 × 10^24^	5.0665 × 10^31^	6.2613 × 10^4^
	Max	2.7949 × 10^44^	1.8218 × 10^55^	1.1983 × 10^26^	2.7913 × 10^52^	2.6824 × 10^14^	2.6965 × 10^22^	3.3101 × 10^35^	6.1316 × 10^42^	3.3447 × 10^14^
	Mean	1.1188 × 10^43^	6.7792 × 10^53^	7.1918 × 10^24^	1.2717 × 10^51^	1.7130 × 10^13^	8.9883 × 10^20^	2.1003 × 10^34^	2.9023 × 10^41^	2.5585 × 10^13^
	Std	5.0853 × 10^43^	3.3270 × 10^54^	2.2580 × 10^25^	5.1032 × 10^51^	4.9641 × 10^13^	4.9231 × 10^21^	7.2555 × 10^34^	1.1238 × 10^42^	7.2185 × 10^13^
	Rank	7	9	4	8	2	3	5	6	1
	*p*-value	3.0199 × 10^−11^	3.0199 × 10^−11^	3.0199 × 10^−11^	3.0199 × 10^−11^	3.5137 × 10^−2^	5.4617 × 10^−9^	3.0199 × 10^−11^	3.0199 × 10^−11^	-
F3	Min	9.3798 × 10^4^	1.6570 × 10^5^	6.7056 × 10^4^	1.5029 × 10^5^	2.5152 × 10^3^	9.7560 × 10^3^	1.2175 × 10^5^	3.2351 × 10^3^	3.7305 × 10^3^
	Max	5.2367 × 10^5^	5.3531 × 10^10^	1.4788 × 10^5^	2.7782 × 10^6^	2.8997 × 10^4^	4.0783 × 10^4^	4.5221 × 10^5^	2.7003 × 10^4^	1.8743 × 10^4^
	Mean	1.1306 × 10^5^	1.9316 × 10^9^	1.1091 × 10^5^	4.0455 × 10^5^	1.2249 × 10^4^	2.1596 × 10^4^	2.6893 × 10^5^	1.1526 × 10^4^	1.0662 × 10^4^
	Std	1.0781 × 10^4^	9.7489 × 10^9^	1.9454 × 10^4^	5.1915 × 10^5^	6.1721 × 10^3^	7.2295 × 10^3^	6.3779 × 10^4^	6.3253 × 10^3^	3.6521 × 10^3^
	Rank	6	9	5	8	2	4	7	3	1
	*p*-value	1.2118 × 10^−12^	1.2118 × 10^−12^	1.2118 × 10^−12^	1.2118 × 10^−12^	NaN	1.2118 × 10^−12^	1.2118 × 10^−12^	NaN	-
F4	Min	5.8946 × 10^2^	1.0314 × 10^4^	4.8507 × 10^2^	8.8614 × 10^3^	4.7389 × 10^2^	4.0953 × 10^2^	6.3226 × 10^2^	4.7040 × 10^2^	4.1566 × 10^2^
	Max	8.2750 × 10^2^	6.5144 × 10^4^	5.6890 × 10^2^	4.5406 × 10^4^	5.2629 × 10^2^	5.3342 × 10^2^	1.3639 × 10^3^	5.2245 × 10^2^	5.2182 × 10^2^
	Mean	7.1050 × 10^2^	4.2604 × 10^4^	5.3124 × 10^2^	2.5276 × 10^4^	4.9765 × 10^2^	5.0183 × 10^2^	9.3728 × 10^2^	4.9125 × 10^2^	4.9033 × 10^2^
	Std	5.9761 × 10^1^	1.2617 × 10^4^	1.8107 × 10^1^	9.5705 × 10^3^	1.4174 × 10^1^	2.3918 × 10^1^	1.8251 × 10^2^	1.8396 × 10^1^	2.4232 × 10^1^
	Rank	6	9	5	8	3	4	7	2	1
	*p*-value	3.0199 × 10^−11^	3.0199 × 10^−11^	6.7220 × 10^−10^	3.0199 × 10^−11^	3.0317 × 10^−2^	1.3345 × 10^−1^	3.0199 × 10^−11^	3.0199 × 10^−11^	-
F5	Min	7.1654 × 10^2^	9.5422 × 10^2^	6.9318 × 10^2^	9.3910 × 10^2^	5.8094 × 10^2^	6.2262 × 10^2^	7.5584 × 10^2^	6.0248 × 10^2^	5.9751 × 10^2^
	Max	7.8028 × 10^2^	1.2844 × 10^3^	7.5963 × 10^2^	1.1767 × 10^3^	7.5104 × 10^2^	7.7320 × 10^2^	1.0409 × 10^3^	7.7262 × 10^2^	7.5371 × 10^2^
	Mean	7.5297 × 10^2^	1.1637 × 10^3^	7.2788 × 10^2^	1.0489 × 10^3^	6.2679 × 10^2^	6.9384 × 10^2^	8.4935 × 10^2^	6.9020 × 10^2^	6.6148 × 10^2^
	Std	1.5245 × 10^1^	7.3984 × 10^1^	1.2938 × 10^1^	6.2624 × 10^1^	3.1612 × 10^1^	3.4506 × 10^1^	6.8712 × 10^1^	4.6749 × 10^1^	4.3148 × 10^1^
	Rank	6	9	3	8	1	5	7	4	2
	*p*-value	3.3386 × 10^−3^	3.0199 × 10^−11^	7.9782 × 10^−2^	3.0199 × 10^−11^	1.6980 × 10^−8^	7.6183 × 10^−1^	7.0881 × 10^−8^	4.0772 × 10^−11^	-
F6	Min	6.0000 × 10^2^	6.9858 × 10^2^	6.0322 × 10^2^	6.8868 × 10^2^	6.0060 × 10^2^	6.4167 × 10^2^	6.5717 × 10^2^	6.0133 × 10^2^	6.0349 × 10^2^
	Max	6.2288 × 10^2^	7.6250 × 10^2^	6.1152 × 10^2^	7.4279 × 10^2^	6.1350 × 10^2^	6.6457 × 10^2^	6.9289 × 10^2^	6.5316 × 10^2^	6.5961 × 10^2^
	Mean	6.0000 × 10^2^	6.2900 × 10^1^	6.0631 × 10^2^	7.1490 × 10^2^	6.0340 × 10^2^	6.5720 × 10^2^	6.7537 × 10^2^	6.2528 × 10^2^	6.2502 × 10^2^
	Std	2.7437 × 10^00^	1.2400 × 10^1^	1.8413 × 10^0^	1.2006 × 10^1^	2.4610 × 10^0^	5.6088 × 10^0^	9.1749 × 10^0^	1.2338 × 10^1^	1.4127 × 10^1^
	Rank	1	7	3	9	2	5	8	4	6
	*p*-value	6.3560 × 10^−5^	3.0199 × 10^−11^	1.3289 × 10^−10^	3.0199 × 10^−11^	4.4440 × 10^−7^	7.3803 × 10^−10^	5.4941 × 10^−11^	9.9186 × 10^−11^	-
F7	Min	9.6084 × 10^2^	3.1096 × 10^3^	9.3278 × 10^2^	2.2940 × 10^3^	8.0722 × 10^2^	9.3742 × 10^2^	1.1594 × 10^3^	8.5152 × 10^2^	8.5189 × 10^2^
	Max	1.0117 × 10^3^	3.8617 × 10^3^	9.9053 × 10^2^	3.4478 × 10^3^	9.7487 × 10^2^	1.1843 × 10^3^	1.4545 × 10^3^	1.2944 × 10^3^	1.2630 × 10^3^
	Mean	9.9282 × 10^2^	4.2000 × 10^1^	9.6627 × 10^2^	2.8176 × 10^3^	8.6422 × 10^2^	1.1061 × 10^3^	1.2986 × 10^3^	1.0147 × 10^3^	1.0224 × 10^3^
	Std	1.2683 × 10^1^	1.2200 × 10^1^	1.2768 × 10^1^	2.9262 × 10^2^	3.9480 × 10^1^	5.0331 × 10^1^	6.7914 × 10^1^	1.2166 × 10^2^	1.1825 × 10^2^
	Rank	3	4	2	9	1	5	8	6	7
	*p*-value	8.5338 × 10^−1^	3.0199 × 10^−11^	4.5146 × 10^−2^	3.0199 × 10^−11^	2.4386 × 10^−9^	2.6015 × 10^−8^	4.9752 × 10^−11^	3.0199 × 10^−11^	-
F8	Min	8.7478 × 10^2^	1.2947 × 10^3^	1.0047 × 10^3^	1.1921 × 10^3^	8.6985 × 10^2^	9.0755 × 10^2^	9.3940 × 10^0^	8.7562 × 10^02^	8.8855 × 10^2^
	Max	1.0846 × 10^3^	1.4828 × 10^3^	1.0568 × 10^3^	1.3960 × 10^3^	9.7051 × 10^2^	1.0308 × 10^3^	1.1676 × 10^3^	1.0159 × 10^3^	1.0030 × 10^3^
	Mean	8.9952 × 10^2^	5.2300 × 10^1^	1.0304 × 10^3^	1.2837 × 10^3^	9.1685 × 10^2^	9.5942 × 10^2^	1.0438 × 10^3^	9.4463 × 10^2^	9.3939 × 10^2^
	Std	6.9538 × 10^0^	1.0200 × 10^1^	1.2740 × 10^1^	5.3439 × 10^1^	3.1005 × 10^1^	3.4689 × 10^1^	4.8060 × 10^1^	3.3363 × 10^1^	2.8957 × 10^1^
	Rank	2	5	6	9	1	7	8	4	3
	*p*-value	3.0199 × 10^−11^	3.0199 × 10^−11^	4.5043 × 10^−11^	3.0199 × 10^−11^	2.0023 × 10^−6^	8.8830 × 10^−1^	3.8202 × 10^−10^	3.0199 × 10^−11^	-
F9	Min	7.0032 × 10^3^	1.9054 × 10^4^	2.0538 × 10^3^	1.4697 × 10^4^	1.9214 × 10^3^	5.1607 × 10^3^	6.7932 × 10^3^	6.3749 × 10^3^	1.9424 × 10^3^
	Max	1.6985 × 10^4^	4.7601 × 10^4^	4.5199 × 10^3^	3.0143 × 10^4^	1.0980 × 10^4^	9.6916 × 10^3^	3.2571 × 10^4^	1.2225 × 10^4^	8.5475 × 10^3^
	Mean	1.1643 × 10^4^	3.7500 × 10^4^	2.7952 × 10^3^	2.4068 × 10^4^	5.2442 × 10^3^	7.6564 × 10^3^	1.3059 × 10^4^	9.7479 × 10^3^	5.1747 × 10^3^
	Std	2.3773 × 10^3^	5.9949 × 10^3^	6.0699 × 10^2^	4.0708 × 10^3^	2.2777 × 10^3^	1.1944 × 10^3^	5.8199 × 10^3^	1.3856 × 10^3^	1.6948 × 10^3^
	Rank	6	9	1	8	3	4	7	5	2
	*p*-value	3.6897 × 10^−11^	3.0199 × 10^−11^	4.1178 × 10^−6^	3.0199 × 10^−11^	6.5204 × 10^−1^	6.0459 × 10^−7^	4.1997 × 10^−10^	1.4643 × 10^−10^	-
F10	Min	8.4132 × 10^3^	9.7867 × 10^3^	7.7355 × 10^3^	8.1999 × 10^3^	3.0770 × 10^3^	4.6398 × 10^3^	5.0641 ×10^3^	3.6012 × 10^3^	2.5686 × 10^3^
	Max	9.8864 × 10^3^	1.1866 × 10^4^	9.3152 × 10^3^	1.0092 × 10^4^	6.3150 × 10^3^	7.0070 × 10^3^	8.6416 × 10^3^	7.2093 × 10^3^	7.7606 × 10^3^
	Mean	9.2739 × 10^3^	1.0854 × 10^4^	8.7408 × 10^3^	9.4494 × 10^3^	4.6711 × 10^3^	5.7831 × 10^3^	6.9687 × 10^3^	4.7415 × 10^3^	5.1107 × 10^3^
	Std	2.8525 × 10^2^	3.4400 × 10^3^	3.7295 × 10^2^	4.5335 × 10^2^	7.2820 × 10^2^	6.1827 × 10^2^	9.2541 × 10^2^	7.1060 × 10^2^	9.3500 × 10^2^
	Rank	7	9	5	8	1	3	6	2	4
	*p*-value	3.0199 × 10^−11^	3.0199 × 10^−11^	3.0199 × 10^−11^	3.0199 × 10^−11^	9.2344 × 10^−1^	8.1465 × 10^−5^	1.0702 × 10^−9^	1.6132 × 10^−10^	-
F11	Min	5.5276 × 10^3^	1.3745 × 10^4^	1.1986 × 10^3^	7.5074 × 10^3^	1.1490 × 10^3^	1.2088 × 10^3^	2.6028 × 10^3^	1.1349 × 10^3^	1.1368 × 10^3^
	Max	1.6185 × 10^4^	2.8377 × 10^5^	1.2875 × 10^3^	5.8373 × 10^4^	1.3760 × 10^3^	1.3374 × 10^3^	1.3561 × 10^4^	1.3641 × 10^3^	1.2840 × 10^3^
	Mean	1.0463 × 10^4^	6.3637 × 10^4^	1.2488 × 10^3^	2.8766 × 10^4^	1.2660 × 10^3^	1.2665 × 10^3^	7.0216 × 10^3^	1.2207 × 10^3^	1.2037 × 10^3^
	Std	2.6622 × 10^3^	5.2876 × 10^4^	2.1263 × 10^1^	1.2367 × 10^4^	5.2560 × 10^1^	2.9995 × 10^1^	2.9811 × 10^3^	5.8135 × 10^1^	4.4210 × 10^1^
	Rank	7	9	2	8	5	4	6	3	1
	*p*-value	3.0199 × 10^−11^	3.0199 × 10^−11^	2.0681 × 10^−2^	3.0199 × 10^−11^	2.2658 × 10^−3^	3.0059 × 10^−4^	3.0199 × 10^−11^	3.0199 × 10^−11^	
F12	Min	2.2161 × 10^8^	1.4479 × 10^10^	1.2600 × 10^6^	5.2363 × 10^9^	4.8730 × 10^5^	2.1766 × 10^6^	3.3151 × 10^7^	2.8406 × 10^4^	7.3759 × 10^4^
	Max	9.3358 × 10^8^	3.9797 × 10^10^	1.0749 × 10^7^	2.6238 × 10^10^	8.5280 × 10^6^	4.2295 × 10^7^	8.9433 × 10^8^	2.3103 × 10^6^	1.5100 × 10^6^
	Mean	4.5281 × 10^8^	2.6199 × 10^10^	4.3229 × 10^6^	1.5373 × 10^10^	2.9180 × 10^6^	1.6871 × 10^7^	2.8065 × 10^8^	4.6619 × 10^5^	3.5144 × 10^5^
	Std	1.5863 × 10^8^	6.5010 × 10^9^	2.4314 × 10^6^	5.1740 × 10^9^	1.9490 × 10^6^	1.0816 × 10^7^	2.2948 × 10^8^	5.0090 × 10^5^	3.1390 × 10^5^
	Rank	7	9	4	8	3	5	6	2	1
	*p*-value	3.0199 × 10^−11^	3.0199 × 10^−11^	3.0199 × 10^−11^	3.0199 × 10^−11^	5.5727 × 10^−10^	3.0199 × 10^−11^	3.0199 × 10^−11^	3.0199 × 10^−11^	-
F13	Min	1.1452 × 10^4^	9.2894 × 10^9^	4.2655 × 10^3^	4.2862 × 10^9^	9.4720 × 10^3^	3.6456 × 10^5^	4.6561 × 10^5^	1.3532 × 10^3^	1.5607 × 10^3^
	Max	2.6848 × 10^7^	4.8535 × 10^10^	2.2415 × 10^4^	2.8201 × 10^10^	7.1220 × 10^4^	4.7730 × 10^6^	8.7062 × 10^6^	5.9713 × 10^4^	6.2857 × 10^4^
	Mean	1.7766 × 10^4^	2.2200 × 10^3^	9.2416 × 10^3^	1.3551 × 10^10^	3.5200 × 10^4^	1.0753 × 10^6^	2.9308 × 10^6^	1.6871 × 10^4^	1.9422 × 10^4^
	Std	7.5745 × 10^3^	1.2200 × 10^2^	3.7397 × 10^3^	5.5841 × 10^9^	2.5470 × 10^4^	7.7927 × 10^5^	2.3216 × 10^6^	1.5084 × 10^4^	1.8341 × 10^4^
	Rank	4	5	1	9	6	7	8	2	3
	*p*-value	3.0199 × 10^−11^	3.0199 × 10^−11^	5.1060 × 10^−1^	3.0199 × 10^−11^	7.6588 × 10^−5^	3.0199 × 10^−11^	3.0199 × 10^−11^	3.0199 × 10^−11^	-
F14	Min	9.6728 × 10^4^	5.2670 × 10^6^	1.4663 × 10^3^	1.7641 × 10^6^	9.4450 × 10^3^	4.9433 × 10^3^	4.7043 × 10^4^	2.9000 × 10^3^	2.8036 × 10^3^
	Max	1.0616 × 10^6^	1.8985 × 10^8^	1.5120 × 10^3^	1.2344 × 10^8^	1.9580 × 10^5^	2.0460 × 10^5^	1.3090 × 10^7^	5.4617 × 10^4^	4.0539 × 10^4^
	Mean	2.0324 × 10^5^	7.3300 × 10^1^	1.4927 × 10^3^	2.5264 × 10^7^	1.0290 × 10^5^	4.4592 × 10^4^	3.3724 × 10^6^	1.6243 × 10^4^	1.1884 × 10^4^
	Std	5.5827 × 10^4^	7.3300 × 10^2^	9.1438 × 10^0^	2.5037 × 10^7^	5.2110 × 10^4^	4.6969 × 10^4^	2.9973 × 10^6^	1.4535 × 10^4^	9.1818 × 10^3^
	Rank	7	5	1	9	6	4	8	3	2
	*p*-value	3.0199 × 10^−11^	3.0199 × 10^−11^	3.0199 × 10^−11^	3.0199 × 10^−11^	7.7725 × 10^−9^	1.6813 × 10^−4^	3.0199 × 10^−11^	6.6955 × 10^−11^	-
F15	Min	2.0459 × 10^3^	1.8897 × 10^9^	1.5964 × 10^3^	1.9734 × 10^8^	1.9910 × 10^3^	5.8591 × 10^4^	8.3870 × 10^4^	1.5717 × 10^3^	1.5505 × 10^3^
	Max	5.5536 × 10^6^	1.3480 × 10^10^	1.7114 × 10^3^	6.2995 ×10^9^	4.3310 × 10^4^	3.3304 × 10^5^	2.2859 × 10^7^	4.0793 × 10^4^	4.2819 × 10^4^
	Mean	8.8916 × 10^3^	5.2400 × 10^3^	1.6582 × 10^3^	2.6018 × 10^9^	2.4960 × 10 ^4^	1.5894 × 10^5^	2.1739 × 10^6^	8.7091 × 10^3^	1.1109 × 10^4^
	Std	3.1246 × 10^3^	7.7000 × 10^2^	2.5041 × 10^1^	1.3775 × 10^9^	1.4300 × 10^4^	6.5396 × 10^4^	4.2818 × 10^6^	9.2998 × 10^3^	1.2935 × 10^4^
	Rank	4	6	1	9	5	7	8	2	3
	*p*-value	3.0199 × 10^−11^	3.0199 × 10^−11^	6.5183 × 10^−9^	3.0199 × 10^−11^	1.7836 × 10^−4^	3.0199 × 10^−11^	3.0199 × 10^−11^	3.0199 × 10^−11^	-
F16	Min	2.0452 × 10^3^	5.8741 × 10^3^	3.1551 × 10^3^	4.8248 × 10^3^	1.7720 × 10^3^	2.7007 × 10^3^	2.4831 × 10^3^	2.1619 × 10^3^	1.9438 × 10^3^
	Max	4.3828 × 10^3^	1.3395 × 10^4^	3.8340 × 10^3^	1.1386 × 10^4^	3.2970 × 10^3^	3.5684 × 10^3^	6.7716 × 10^3^	3.2044 × 10^3^	3.2119 × 10^3^
	Mean	4.0016 × 10^3^	5.5600 × 10^3^	3.5677 × 10^3^	6.7991 × 10^3^	2.5070 × 10^3^	3.1605 × 10^3^	4.2004 × 10^3^	2.6004 × 10^3^	2.4746 × 10^3^
	Std	2.2691 × 10^2^	2.2000 × 10^2^	1.8219 × 10^2^	1.3622 × 10^3^	3.3580 × 10^2^	2.4959 × 10^2^	8.2898 × 10^2^	3.1577 × 10^2^	2.7366 × 10^2^
	Rank	4	8	5	9	2	6	7	3	1
	*p*-value	3.0199 × 10^−11^	3.0199 × 10^−11^	3.0199 × 10^−11^	3.0199 × 10^−11^	9.7052 × 10^−01^	1.2023 × 10^−08^	8.9934 × 10^−11^	3.0199 × 10^−11^	-
F17	Min	1.8441 × 10^3^	4.6659 × 10^3^	2.1103 × 10^3^	3.3244 × 10^3^	1.8040 × 10^3^	2.0717 × 10^3^	2.2412 × 10^3^	1.7956 × 10^3^	1.7925 × 10^3^
	Max	3.1530 × 10^3^	3.4404 × 10^5^	2.6988 × 10^3^	3.2925 × 10^4^	2.7230 × 10^3^	3.3136 × 10^3^	3.2823 × 10^3^	2.6236 × 10^3^	2.5946 × 10^3^
	Mean	2.9495 × 10^3^	4.7341 × 10^4^	2.4235 × 10^3^	1.0638 × 10^4^	2.2450 × 10^3^	2.5941 × 10^3^	2.7821 × 10^3^	2.1502 × 10^3^	2.1664 × 10^3^
	Std	1.6006 × 10^2^	2.6600 × 10^3^	1.4637 × 10^2^	7.7982 × 10^3^	2.1920 × 10^2^	2.7539 × 10^2^	2.7887 × 10^2^	2.0232 × 10^2^	1.8561 × 10^2^
	Rank	5	9	3	8	4	6	7	2	1
	*p*-value	4.5043 × 10^−11^	3.0199 × 10^−11^	1.1077 × 10^−06^	3.0199 × 10^−11^	2.4157 × 10^−2^	7.6588 × 10^−5^	1.5581 × 10^−8^	2.3897 × 10^−8^	-
F18	Min	1.3677 × 10^5^	4.1987 × 10^7^	4.7817 × 10^3^	1.4347 × 10^7^	1.2430 × 10^5^	9.9131 × 10^4^	3.1313 × 10^5^	4.1933 × 10^4^	4.6072 × 10^4^
	Max	3.8236 × 10^7^	1.5215 × 10^9^	4.6879 × 10^4^	5.1200 × 10^8^	4.2940 × 10^6^	3.3343 × 10^6^	6.0586 × 10^7^	1.3249 × 10^6^	1.8493 × 10^6^
	Mean	3.0928 × 10^5^	1.6000 × 10^3^	1.8993 × 10^4^	1.2777 × 10^8^	1.4650 × 10^6^	6.1749 × 10^5^	8.8647 × 10^6^	2.7455 × 10^5^	2.8187 × 10^5^
	Std	9.6714 × 10^4^	5.7300 × 10^2^	1.0976 × 10^4^	1.2244 × 10^8^	1.2390 × 10^6^	6.5543 × 10^5^	1.3895 × 10^7^	2.6898 × 10^5^	3.7817 × 10^5^
	Rank	4	5	1	9	7	6	8	2	3
	*p*-value	3.0199 × 10^−11^	3.0199 × 10^−11^	4.5043 × 10^−11^	3.0199 × 10^−11^	1.0105 × 10^−8^	1.5014 × 10^−2^	1.6132 × 10^−10^	8.1014 × 10^−10^	-
F19	Min	3.3711 × 10^3^	2.4416 × 10^9^	1.9460 × 10^3^	1.3628 × 10^9^	7.1370 × 10^3^	1.4973 × 10^5^	4.2547 × 10^4^	1.9200 × 10^3^	1.9943 × 10^3^
	Max	4.6006 × 10^5^	2.3046 × 10^10^	1.9999 × 10^3^	6.0346 × 10^9^	5.6570 × 10^4^	2.1529 × 10^6^	3.1903 × 10^7^	5.6153 × 10^4^	4.9774 × 10^4^
	Mean	8.5326 × 10^3^	3.3200 × 10^2^	1.9642 × 10^3^	2.9205 × 10^9^	3.8150 × 10^4^	8.7076 × 10^5^	1.1132 × 10^7^	1.1733 × 10^4^	1.0513 × 10^4^
	Std	5.0755 × 10^3^	1.2200 × 10^3^	1.1177 × 10^1^	1.1896 × 10^9^	1.9450 × 10^4^	5.4963 × 10^5^	7.9663 × 10^6^	1.4443 × 10^4^	1.1281 × 10^4^
	Rank	4	5	1	9	6	7	8	3	2
	*p*-value	1.6132 × 10^−10^	3.0199 × 10^−11^	5.5727 × 10^−10^	3.0199 × 10^−11^	8.6844 × 10^−3^	3.0199 × 10^−11^	3.0199 × 10^−11^	3.0199 × 10^−11^	-
F20	Min	2.1506 × 10^3^	3.4140 × 10^3^	2.6418 × 10^3^	2.9675 × 10^3^	2.2980 × 10^3^	2.3455 × 10^3^	2.5271 × 10^3^	2.1960 × 10^3^	2.1806 × 10^3^
	Max	3.3109 × 10^3^	4.3081 × 10^3^	3.0783 × 10^3^	3.6499 × 10^3^	2.7500 × 10^3^	3.1023 × 10^3^	3.3016 × 10^3^	2.8994 × 10^3^	2.9051 × 10^3^
	Mean	2.2816 × 10^3^	3.2200 × 10^3^	2.8286 × 10^3^	3.3611 × 10^3^	2.5160 × 10^3^	2.7642 × 10^3^	2.9118 × 10^3^	2.4544 × 10^3^	2.5069 × 10^3^
	Std	4.5941 × 10^1^	4.2300 × 10^2^	1.1178 × 10^2^	1.5474 × 10^2^	1.4400 × 10^2^	2.0481 × 10^2^	2.3447 × 10^2^	1.8973 × 10^2^	1.9487 × 10^2^
	Rank	1	9	5	8	2	6	7	3	4
	*p*-value	4.9752 × 10^−11^	3.0199 × 10^−11^	7.3803 × 10^−10^	3.0199 × 10^−11^	1.3272 × 10^−2^	2.6784 × 10^−6^	1.3289 × 10^−10^	1.2023 × 10^−8^	-
F21	Min	2.4998 × 10^3^	2.7335 × 10^3^	2.4938 × 10^3^	2.6432 × 10^3^	2.3610 × 10^3^	2.4422 × 10^3^	2.5117 × 10^3^	2.3667 × 10^3^	2.3609 × 10^3^
	Max	2.5791 × 10^3^	3.0507 × 10^3^	2.5555 × 10^3^	2.8797 × 10^3^	2.4870 × 10^3^	2.6160 × 10^3^	2.7173 × 10^3^	2.5234 × 10^3^	2.5046 × 10^3^
	Mean	2.3037 × 10^3^	2.9379 × 10^3^	2.5278 × 10^3^	2.7886 × 10^3^	2.4090 × 10^3^	2.5320 × 10^3^	2.6246 × 10^3^	2.4281 × 10^3^	2.4234 × 10^3^
	Std	7.5583 × 10^1^	7.9959 × 10^1^	1.4103 × 10^1^	5.9467 × 10^1^	2.4530 × 10^1^	4.4806 × 10^1^	6.3342 × 10^1^	4.2821 × 10^1^	2.9822 × 10^1^
	Rank	5	9	4	8	1	6	7	3	2
	*p*-value	4.5043 × 10^−11^	3.0199 × 10^−11^	2.3715 × 10^−10^	3.0199 × 10^−11^	6.7350 × 10^−1^	2.0338 × 10^−9^	4.0772 × 10^−11^	3.0199 × 10^−11^	-
F22	Min	2.3000 × 10^3^	1.0306 × 10^4^	2.3168 × 10^3^	7.9256 × 10^3^	2.3010 × 10^3^	2.3236 × 10^3^	2.8013 × 10^3^	2.3000 × 10^3^	2.3000 × 10^3^
	Max	1.0900 × 10^4^	1.3120 × 10^4^	2.3262 × 10^3^	1.2211 × 10^4^	7.7140 × 10^3^	8.5579 × 10^3^	1.0547 × 10^4^	7.7688 × 10^3^	6.4637 × 10^3^
	Mean	2.3128 × 10^3^	4.3300 × 10^2^	2.3211 × 10^3^	1.0590 × 10^4^	5.7980 × 10^3^	5.9502 × 10^3^	8.1343 × 10^3^	3.1031 × 10^3^	2.4397 × 10^3^
	Std	3.5097 × 10^0^	2.3300 × 10^3^	2.1808 × 10^0^	8.8398 × 10^2^	1.1790 × 10^3^	2.1662 × 10^3^	1.6086 × 10^3^	1.8419 × 10^3^	7.6001 × 10^2^
	Rank	3	8	1	9	4	6	7	5	2
	*p*-value	3.3384 × 10^−11^	3.0199 × 10^−11^	8.4848 × 10^−9^	3.0199 × 10^−11^	5.4617 × 10^−9^	6.7220 × 10^−10^	9.9186 × 10^−11^	1.9568 × 10^−10^	-
F23	Min	2.8749 × 10^3^	3.3073 × 10^3^	2.8510 × 10^3^	3.2778 × 10^3^	2.7238 × 10^3^	3.0471 × 10^3^	2.9423 × 10^3^	2.6967 × 10^3^	2.7000 × 10^3^
	Max	2.9480 × 10^3^	4.2982 × 10^3^	2.9063 × 10^3^	3.7566 × 10^3^	2.8054 × 10^3^	3.6307 × 10^3^	3.3612 × 10^3^	2.9593 × 10^3^	2.8401 × 10^3^
	Mean	2.9172 × 10^3^	3.8783 × 10^3^	2.8843 × 10^3^	3.5453 × 10^3^	2.7566 × 10^3^	3.3266 × 10^3^	3.1063 × 10^3^	2.7818 × 10^3^	2.7615 × 10^3^
	Std	1.8843 × 10^1^	2.3000 × 10^3^	1.4124 × 10^1^	1.3473 × 10^2^	1.9185 × 10^1^	1.5349 × 10^2^	1.0442 × 10^2^	4.5888 × 10^1^	3.6622 × 10^1^
	Rank	5	9	3	8	1	7	6	4	2
	*p*-value	4.9752 × 10^−11^	3.0199 × 10^−11^	3.8202 × 10^−10^	3.0199 × 10^−11^	4.2039 × 10^−1^	3.0199 × 10^−11^	3.0199 × 10^−11^	3.0199 × 10^−11^	-
F24	Min	3.0675 × 10^3^	3.8413 × 10^3^	3.0087 × 10^3^	3.4658 × 10^3^	2.8850 × 10^3^	3.1984 × 10^3^	3.0610 × 10^3^	2.8646 × 10^3^	2.8767 × 10^3^
	Max	3.1290 × 10^3^	4.8371 × 10^3^	3.0811 × 10^3^	4.4138 × 10^3^	3.0170 × 10^3^	3.6435 × 10^3^	3.4669 × 10^3^	3.0880 × 10^3^	2.9916 × 10^3^
	Mean	3.0956 × 10^3^	4.3577 × 10^3^	3.0513 × 10^3^	3.7671 × 10^3^	2.9420 × 10^3^	3.3670 × 10^3^	3.2159 × 10^3^	2.9248 × 10^3^	2.9276 × 10^3^
	Std	1.7187 × 10^2^	6.3300 × 10^2^	2.6623 × 10^2^	2.3032 × 10^2^	3.4050 × 10^1^	1.1382 × 10^2^	1.0195 × 10^2^	4.3333 × 10^1^	2.6834 × 10^1^
	Rank	6	9	4	8	3	7	5	2	1
	*p*-value	3.0199 × 10^−11^	3.0199 × 10^−11^	6.6955 × 10^−11^	3.0199 × 10^−11^	3.9167 × 10^−2^	3.0199 × 10^−11^	3.3384 × 10^−11^	3.0199 × 10^−11^	-
F25	Min	2.8837 × 10^3^	1.1399 × 10^4^	2.8914 × 10^3^	6.2271 × 10^3^	2.8840 × 10^3^	2.8864 × 10^3^	3.0208 × 10^3^	2.8836 × 10^3^	2.8836 × 10^3^
	Max	3.0289 × 10^3^	2.5458 × 10^4^	2.9291 × 10^3^	1.5988 × 10^4^	2.9270 × 10^3^	2.9518 × 10^3^	3.2495 × 10^3^	2.9429 × 10^3^	2.9409 × 10^3^
	Mean	2.9808 × 10^3^	1.8598 × 10^4^	2.9018 × 10^3^	1.1112 × 10^4^	2.8957 × 10^3^	2.9090 × 10^3^	3.1302 × 10^3^	2.8939 × 10^3^	2.8984 × 10^3^
	Std	2.0681 × 10^1^	4.2200 × 10^1^	8.3757 × 10^0^	2.4224 × 10^3^	1.0550 × 10^1^	2.0669 × 10^1^	5.8141 × 10^1^	1.5232 × 10^1^	1.9458 × 10^1^
	Rank	6	9	1	8	4	5	7	2	3
	*p*-value	3.0199 × 10^−11^	3.0199 × 10^−11^	3.8307 × 10^−5^	3.0199 × 10^−11^	5.6922 × 10^−1^	2.0023 × 10^−6^	3.0199 × 10^−11^	3.0199 × 10^−11^	-
F26	Min	2.8361 × 10^3^	1.1704 × 10^4^	5.3538 × 10^3^	9.6453 × 10^3^	4.4010 × 10^3^	2.8905 × 10^3^	4.1644 × 10^3^	2.8000 × 10^3^	2.8000 × 10^3^
	Max	6.1479 × 10^3^	2.2693 × 10^4^	6.0782 × 10^3^	1.5103 × 10^4^	5.2560 × 10^3^	1.0225 × 10^4^	1.0138 × 10^4^	7.8492 × 10^3^	7.7356 × 10^3^
	Mean	2.9114 × 10^3^	3.4400 × 10^1^	5.8138 × 10^3^	1.2221 × 10^4^	4.7580 × 10^3^	7.3677 × 10^3^	8.1429 × 10^3^	5.1373 × 10^3^	5.1514 × 10^3^
	Std	2.8268 × 10^1^	2.5500 × 10^2^	1.4751 × 10^2^	1.2602 × 10^3^	2.1090 × 10^2^	1.9198 × 10^3^	1.1711 × 10^3^	1.4116 × 10^3^	1.4729 × 10^3^
	Rank	1	6	3	9	2	8	7	4	5
	*p*-value	4.6390 × 10^−5^	3.0199 × 10^−11^	9.7917 × 10^−5^	3.0199 × 10^−11^	6.7350 × 10^−1^	2.1959 × 10^−7^	6.0658 × 10^−11^	3.0199 × 10^−11^	-
F27	Min	3.2035 × 10^3^	4.3757 × 10^3^	3.2797 × 10^3^	3.9060 × 10^3^	3.1960 × 10^3^	3.3824 × 10^3^	3.3121 × 10^3^	3.2117 × 10^3^	3.2042 × 10^3^
	Max	3.2000 × 10^3^	6.4912 × 10^3^	3.3598 × 10^3^	5.1682 × 10^3^	3.2650 × 10^3^	4.4337 × 10^3^	3.8718 × 10^3^	3.3703 × 10^3^	3.3554 × 10^3^
	Mean	3.2068 × 10^3^	6.3300 × 10^2^	3.3131 × 10^3^	4.4317 × 10^3^	3.2250 × 10^3^	3.7969 × 10^3^	3.4806 × 10^3^	3.2394 × 10^3^	3.2369 × 10^3^
	Std	4.7499 × 10^0^	8.2200 × 10^1^	2.3424 × 10^1^	3.0325 × 10^2^	1.7090 × 10^1^	2.7048 × 10^2^	1.3175 × 10^2^	3.0071 × 10^1^	2.8526 × 10^1^
	Rank	1	6	4	9	2	8	7	5	3
	*p*-value	3.0199 × 10^−11^	3.0199 × 10^−11^	3.6897 × 10^−11^	3.0199 × 10^−11^	6.6273 × 10^−1^	3.0199 × 10^−11^	3.6897 × 10^−11^	3.0199 × 10^−11^	-
F28	Min	3.1485 × 10^3^	9.7515 × 10^3^	3.2360 × 10^3^	7.3217 × 10^3^	3.2010 × 10^3^	3.2096 × 10^3^	3.4433 × 10^3^	3.1774 × 10^3^	3.1475 × 10^3^
	Max	3.3000 × 10^3^	1.7571 × 10^4^	3.3029 × 10^3^	1.2710 × 10^4^	3.3490 × 10^3^	3.2792 × 10^3^	4.0095 × 10^3^	3.2612 × 10^3^	3.2628 × 10^3^
	Mean	3.2013 × 10^3^	8.3300 × 10^2^	3.2658 × 10^3^	9.5271 × 10^3^	3.2520 × 10^3^	3.2426 × 10^3^	3.6372 × 10^3^	3.2101 × 10^3^	3.2066 × 10^3^
	Std	1.0859 × 10^1^	3.2200 × 10^3^	1.9187 × 10^1^	1.3320 × 10^3^	3.5840 × 10^1^	2.3085 × 10^1^	1.2677 × 10^2^	1.9640 × 10^1^	2.2023 × 10^1^
	Rank	1	7	5	9	6	4	8	3	2
	*p*-value	3.0199 × 10^−11^	3.0199 × 10^−11^	9.2603 × 10^−9^	3.0199 × 10^−11^	1.7479 × 10^−5^	7.1988 × 10^−5^	3.0199 × 10^−11^	3.0199 × 10^−11^	-
F29	Min	4.4928 × 10^3^	6.6630 × 10^3^	4.1331 × 10^3^	6.5491 × 10^3^	3.5400 × 10^3^	3.9504 × 10^3^	4.4103 × 10^3^	3.3856 × 10^3^	3.5042 × 10^3^
	Max	5.3740 × 10^3^	1.2556 × 10^5^	4.8043 × 10^3^	2.5381 × 10^4^	4.2920 × 10^3^	5.2354 × 10^3^	6.4875 × 10^3^	4.1276 × 10^3^	4.1586 × 10^3^
	Mean	4.9700 × 10^3^	7.3300 × 10^3^	4.4994 × 10^3^	1.2062 × 10^4^	3.9020 × 10^3^	4.5567 × 10^3^	5.3922 × 10^3^	3.8356 × 10^3^	3.8396 × 10^3^
	Std	2.4081 × 10^2^	3.4400 × 10^2^	1.6904 × 10^2^	4.8332 × 10^3^	1.7880 × 10^2^	3.1462 × 10^2^	5.5518 × 10^2^	2.0514 × 10^2^	1.7926 × 10^2^
	Rank	6	8	4	9	3	5	7	1	2
	*p*-value	3.0199 × 10^−11^	3.0199 × 10^−11^	4.0772 × 10^−11^	3.0199 × 10^−11^	3.2651 × 10^−2^	2.8716 × 10^−10^	3.0199 × 10^−11^	3.0199 × 10^−11^	-
F30	Min	1.2719 × 10^4^	9.3579 × 10^8^	3.5700 × 10^4^	7.2064 × 10^8^	8.8130 × 10^3^	5.0064 × 10^5^	2.9098 × 10^6^	5.3023 × 10^3^	5.6202 × 10^3^
	Max	1.3080 × 10^6^	6.4592 × 10^9^	3.0691 × 10^5^	5.4165 × 10^9^	6.0320 × 10^4^	1.0031 × 10^7^	2.0458 × 10^8^	1.9861 × 10^4^	1.6952 × 10^4^
	Mean	2.1358 × 10^4^	3.3295 × 10^9^	1.0250 × 10^5^	2.4729 × 10^9^	2.4640 × 10^4^	4.3834 × 10^6^	5.3564 × 10^7^	1.0142 × 10^4^	9.4990 × 10^3^
	Std	5.9215 × 10^3^	1.4214 × 10^9^	6.2618 × 10^4^	1.1582 × 10^9^	1.0280 × 10^4^	2.3051 × 10^6^	5.4071 × 10^7^	3.9579 × 10^3^	3.0359 × 10^3^
	Rank	4	9	5	8	3	6	7	2	1
	*p*-value	3.0199 × 10^−11^	3.0199 × 10^−11^	3.0199 × 10^−11^	3.0199 × 10^−11^	3.1589 × 10^−10^	3.0199 × 10^−11^	3.0199 × 10^−11^	3.0199 × 10^−11^	-
+/=/−		29/0/1	30/0/0	28/0/2	30/0/0	21/0/9	27/0/3	30/0/0	29/0/1	-

**Table 6:** Comparison of results obtained—CEC2017 benchmark functions (50D).

Function		ABC [43]	ACO	DE	GA	SMA [50]	PSO [50]	WOA	MRFO [50]	AMRFOCS
F1	Min	2.1737 × 10^10^	2.2302 × 10^11^	3.7780 × 10^8^	1.7644 × 10^11^	2.8025 × 10^6^	4.4042 × 10^7^	1.2604 × 10^10^	7.2939 × 10^4^	3.6432 × 10^4^
	Max	5.3757 × 10^10^	3.1622 × 10^11^	2.2735 × 10^9^	2.5294 × 10^11^	1.2339 × 10^7^	2.1058 × 10^9^	2.8191 × 10^10^	7.0300 × 10^6^	3.1240 × 10^6^
	Mean	3.6270 × 10^10^	2.6670 × 10^11^	8.8973 × 10^8^	2.1598 × 10^11^	6.2106 × 10^6^	5.6369 × 10^8^	2.1789 × 10^10^	4.8755 × 10^5^	3.9104 × 10^5^
	Std	8.0415 × 10^9^	2.0681 × 10^10^	5.0840 × 10^8^	2.4075 × 10^10^	2.2142 × 10^6^	6.5264 × 10^8^	4.5794 × 10^9^	1.2467 × 10^6^	6.9240 × 10^5^
	Rank	7	9	5	8	3	4	6	2	1
	*p*-value	3.0199 × 10^−11^	3.0199 × 10^−11^	3.0199 × 10^−11^	3.0199 × 10^−11^	3.6897 × 10^−11^	3.0199 × 10^−11^	3.0199 × 10^−11^	3.0199 × 10^−11^	-
F2	Min	1.5809 × 10^75^	8.2101 × 10^82^	4.0980 × 10^43^	1.1236 × 10^80^	1.5879 × 10^25^	2.8225 × 10^20^	1.5711 × 10^64^	1.2697 × 10^64^	3.1012 × 10^25^
	Max	3.4176 × 10^83^	5.5082 × 10^96^	1.1547 × 10^56^	8.4006 × 10^92^	2.2968 × 10^38^	2.6945 × 10^52^	2.3354 × 10^84^	1.0921 × 10^83^	4.2615 × 10^40^
	Mean	2.5029 × 10^82^	1.8541 × 10^95^	4.7927 × 10^54^	5.3503 × 10^91^	8.8177 × 10^36^	8.9816 × 10^50^	7.7881 × 10^82^	3.6479 × 10^81^	1.4390 × 10^39^
	Std	7.2547 × 10^82^	1.0053 × 10^96^	2.1507 × 10^55^	2.0042 × 10^92^	4.2031 × 10^37^	4.9194 × 10^51^	4.2638 × 10^83^	1.9937 × 10^82^	7.7774 × 10^39^
	Rank	7	9	4	8	1	3	6	5	2
	*p*-value	3.0199 × 10^−11^	3.0199 × 10^−11^	4.0772 × 10^−11^	3.0199 × 10^−11^	8.1875 × 10^−1^	8.3520 × 10^−8^	3.0199 × 10^−11^	3.0199 × 10^−11^	-
F3	Min	1.8730 × 10^5^	5.1261 × 10^5^	1.7665 × 10^5^	3.0514 × 10^5^	7.7718 × 10^4^	9.2693 × 10^4^	1.9486 × 10^5^	1.0899 × 10^5^	9.0521 × 10^4^
	Max	1.8842 × 10^6^	3.9258 × 10^12^	3.2630 × 10^5^	7.5046 × 10^8^	3.5256 × 10^5^	2.2562 × 10^5^	5.7027 × 10^5^	2.6159 × 10^5^	2.2619 × 10^5^
	Mean	2.2788 × 10^5^	3.6318 × 10^11^	2.6879 × 10^5^	3.4493 × 10^7^	3.0052 × 10^2^	1.1823 × 10^4^	3.3277 × 10^5^	3.0061 × 10^2^	1.6057 × 10^5^
	Std	1.8593 × 10^4^	9.4573 × 10^11^	3.8977 × 10^4^	1.3871 × 10^8^	3.3306 × 10^−1^	3.6550 × 10^3^	1.0007 × 10^5^	1.4242 × 10^0^	3.1640 × 10^4^
	Rank	6	9	5	8	1	2	7	3	4
	*p*-value	3.0199 × 10^−11^	3.0199 × 10^−11^	3.6897 × 10^−11^	3.0199 × 10^−11^	3.0317 × 10^−2^	8.0727 × 10^−1^	8.1527 × 10^−11^	2.8314 × 10^−8^	-
F4	Min	4.2924 × 10^2^	6.7079 × 10^4^	6.7833 × 10^2^	5.4906 × 10^4^	5.2565 × 10^2^	4.8158 × 10^2^	2.8058 × 10^3^	4.4762 × 10^2^	4.3289 × 10^2^
	Max	3.1194 × 10^4^	1.4920 × 10^5^	9.2950 × 10^2^	1.1222 × 10^5^	7.5166 × 10^2^	7.4709 × 10^2^	7.8817 × 10^3^	8.1191 × 10^2^	7.2753 × 10^2^
	Mean	4.5806 × 10^2^	1.1460 × 10^5^	7.8814 × 10^2^	8.2777 × 10^4^	5.4940 × 10^2^	6.9121 × 10^2^	4.8658 × 10^3^	4.6152 × 10^2^	5.7350 × 10^2^
	Std	1.5587 × 10^1^	2.1693 × 10^4^	6.4800 × 10^1^	1.8160 × 10^4^	5.3517 × 10^1^	7.7460 × 10^1^	1.2178 × 10^3^	4.5205 × 10^1^	5.3578 × 10^1^
	Rank	1	9	6	8	4	5	7	2	3
	*p*-value	3.0199 × 10^−11^	3.0199 × 10^−11^	3.6897 × 10^−11^	3.0199 × 10^−11^	4.2067 × 10^−02^	2.8913 × 10^−03^	3.0199 × 10^−11^	3.0199 × 10^−11^	-
F5	Min	6.7622 × 10^2^	1.4434 × 10^3^	9.1209 × 10^2^	1.3227 × 10^3^	6.6596 × 10^2^	7.8626 × 10^2^	1.0008 × 10^3^	7.1991 × 10^2^	7.4477 × 10^2^
	Max	1.2094 × 10^3^	1.8792 × 10^3^	1.0089 × 10^3^	1.7771 × 10^3^	8.8866 × 10^2^	9.5048 × 10^2^	1.3249 × 10^3^	9.3479 × 10^2^	9.1489 × 10^2^
	Mean	7.1659 × 10^2^	1.6872 × 10^3^	9.4975 × 10^2^	1.5792 × 10^3^	7.0992 × 10^2^	6.3764 × 10^2^	1.1352 × 10^3^	8.2425 × 10^2^	8.3384 × 10^2^
	Std	3.0539 × 10^1^	1.1129 × 10^2^	2.4416 × 10^1^	1.0960 × 10^2^	4.3070 × 10^1^	2.9924 × 10^1^	8.3373 × 10^1^	3.9911 × 10^1^	3.6296 × 10^1^
	Rank	3	9	6	8	1	2	7	4	5
	*p*-value	3.0199 × 10^−11^	3.0199 × 10^−11^	3.3384 × 10^−11^	3.0199 × 10^−11^	8.3146 × 10^−3^	4.1191 × 10^−1^	3.0199 × 10^−11^	3.0199 × 10^−11^	-
F6	Min	6.0000 × 10^2^	7.3948 × 10^2^	6.0963 × 10^2^	7.1424 × 10^2^	6.1911 × 10^2^	6.5547 × 10^2^	6.8136 × 10^2^	6.2359 × 10^2^	6.2709 × 10^2^
	Max	7.0165 × 10^2^	7.7541 × 10^2^	6.2515 × 10^2^	7.6016 × 10^2^	6.6413 × 10^2^	6.8208 × 10^2^	7.3238 × 10^2^	6.7067 × 10^2^	6.6725 × 10^2^
	Mean	6.0000 × 10^2^	7.5660 × 10^2^	6.1498 × 10^2^	7.4094 × 10^2^	6.0575 × 10^2^	6.4765 × 10^2^	7.0095 × 10^2^	6.0121 × 10^2^	6.4814 × 10^2^
	Std	3.8755 ×10^−13^	8.8646 × 10^0^	3.8880 × 10^0^	1.0274 × 10^1^	2.2689 × 10^0^	1.1227 × 10^1^	1.2176 × 10^1^	7.6182 ×10^−1^	1.0358 × 10^1^
	Rank	1	9	2	7	3	6	8	4	5
	*p*-value	3.0199 × 10^−11^	3.0199 × 10^−11^	3.0199 × 10^−11^	3.0199 × 10^−11^	5.1060 × 10^−01^	9.9186 × 10^−11^	3.0199 × 10^−11^	3.0199 × 10^−11^	-
F7	Min	9.2657 × 10^2^	4.3362 × 10^3^	1.1683 × 10^3^	4.7100 × 10^3^	1.0340 × 10^3^	1.6035 × 10^3^	1.7225 × 10^3^	1.2525 × 10^3^	1.1067 × 10^3^
	Max	2.0399 × 10^3^	7.0315 × 10^3^	1.2890 × 10^3^	5.9726 × 10^3^	1.3204 × 10^3^	1.9375 × 10^3^	2.0341 × 10^3^	1.8252 × 10^3^	1.7588 × 10^3^
	Mean	9.3679 × 10^2^	6.0513 × 10^3^	1.2322 × 10^3^	5.4172 × 10^3^	9.8578 × 10^2^	9.4995 × 10^2^	1.8734 × 10^3^	1.4977 × 10^3^	1.4712 × 10^3^
	Std	1.3669 × 10^1^	5.0609 × 10^2^	3.0198 × 10^1^	3.6143 × 10^2^	4.6819 × 10^1^	4.9071 × 10^1^	7.8377 × 10^1^	1.3581 × 10^2^	1.5023 × 10^2^
	Rank	1	9	3	8	2	4	7	6	5
	*p*-value	1.8731 × 10^−7^	3.0199 × 10^−11^	1.3111 × 10^−8^	3.0199 × 10^−11^	5.5727 × 10^−10^	1.6351 × 10^−5^	4.6159 × 10^−10^	5.4941 × 10^−11^	-
F8	Min	9.5307 × 10^2^	1.8134 × 10^3^	1.1621 × 10^3^	1.7187 × 10^3^	1.0106 × 10^3^	1.1057 × 10^3^	1.2746 × 10^3^	1.0109 × 10^3^	1.0667 × 10^3^
	Max	1.5179 × 10^3^	2.1794 × 10^3^	1.2895 × 10^3^	2.0365 × 10^3^	1.2399 × 10^3^	1.2817 × 10^3^	1.6484 × 10^3^	1.2428 × 10^3^	1.2248 × 10^3^
	Mean	1.0207 × 10^3^	2.0123 × 10^3^	1.2439 × 10^3^	1.8588 × 10^3^	9.8827 × 10^2^	9.4820 × 10^2^	1.3998 × 10^3^	1.1382 × 10^3^	1.1408 × 10^3^
	Std	2.2909 × 10^1^	8.7077 × 10^1^	2.5499 × 10^1^	8.7226 × 10^1^	4.5826 × 10^1^	2.3206 × 10^1^	7.1058 × 10^1^	4.4933 × 10^1^	4.0742 × 10^1^
	Rank	1	9	6	8	2	3	7	5	4
	*p*-value	3.0199 × 10^−11^	3.0199 × 10^−11^	1.7769 × 10^−10^	3.0199 × 10^−11^	7.6171 × 10^−3^	3.6439 × 10^−2^	3.0199 × 10^−11^	3.0199 × 10^−11^	-
F9	Min	4.8233 × 10^4^	7.2918 × 10^4^	5.8535 × 10^3^	5.7641 × 10^4^	9.4579 × 10^3^	2.4979 × 10^4^	2.6835 × 10^4^	2.9374 × 10^4^	1.2546 × 10^4^
	Max	9.0949 × 10^4^	1.2119 × 10^5^	2.1322 × 10^4^	1.0598 × 10^5^	2.9632 × 10^4^	4.2548 × 10^4^	7.4146 × 10^4^	4.5420 × 10^4^	3.5896 × 10^4^
	Mean	7.3802 × 10^4^	9.9799 × 10^4^	1.3925 × 10^4^	8.2871 × 10^4^	1.8497 × 10^4^	3.4493 × 10^4^	3.9735 × 10^4^	3.6275 × 10^4^	2.3481 × 10^4^
	Std	9.4844 × 10^3^	1.2560 × 10^4^	3.8673 × 10^3^	1.0137 × 10^4^	4.4405 × 10^3^	4.1921 × 10^3^	9.8762 × 10^3^	3.9604 × 10^3^	5.8765 × 10^3^
	Rank	7	9	1	8	2	4	6	5	3
	*p*-value	3.6897 × 10^−11^	3.0199 × 10^−11^	4.1178 × 10^−6^	3.0199 × 10^−11^	6.5204 × 10^−6^	6.0459 × 10^−7^	4.1997 × 10^−10^	1.4643 × 10^−10^	-
F10	Min	1.4934 × 10^4^	1.7224 × 10^4^	1.4494 × 10^4^	1.5148 × 10^4^	6.2925 × 10^3^	7.0489 × 10^3^	1.1265 × 10^4^	6.1837 × 10^3^	5.5321 × 10^3^
	Max	1.6715 × 10^4^	1.9414 × 10^4^	1.6160 × 10^4^	1.8002 × 10^4^	9.8385 × 10^3^	1.0939 × 10^4^	1.5006 × 10^4^	9.6680 × 10^3^	1.1909 × 10^4^
	Mean	1.6150 × 10^4^	1.8362 × 10^4^	1.5510 × 10^4^	1.6538 × 10^4^	7.3335 × 10^3^	6.9984 × 10^3^	1.3376 × 10^4^	7.4781 × 10^3^	7.9009 × 10^3^
	Std	5.6697 × 10^2^	5.1518 × 10^2^	4.2387 × 10^2^	5.5881 × 10^2^	8.1754 × 10^2^	1.3399 × 10^3^	9.6279 × 10^2^	8.6425 × 10^2^	1.2515 × 10^3^
	Rank	7	9	4	8	5	3	6	2	1
	*p*-value	3.0199 × 10^−11^	3.0199 × 10^−11^	3.0199 × 10^−11^	3.0199 × 10^−11^	3.1830 × 10^−1^	7.2208 × 10^−6^	1.9568 × 10^−10^	8.9934 × 10^−11^	-
F11	Min	2.0214 × 10^3^	6.3864 × 10^4^	1.4631 × 10^3^	4.1450 × 10^4^	1.2675 × 10^3^	1.3884 × 10^3^	4.7453 × 10^3^	1.2529 × 10^3^	1.2173 × 10^3^
	Max	1.1219 × 10^5^	1.2177 × 10^8^	2.1360 × 10^3^	3.4811 × 10^5^	1.6147 × 10^3^	1.6474 × 10^3^	1.2710 × 10^4^	1.4930 × 10^3^	1.5044 × 10^3^
	Mean	4.5105 × 10^3^	7.8482 × 10^6^	1.6211 × 10^3^	1.1678 × 10^5^	1.3906 × 10^3^	1.3255 × 10^3^	8.4656 × 10^3^	1.3531 × 10^3^	1.3283 × 10^3^
	Std	1.4754 × 10^3^	2.4554 × 10^7^	1.6887 × 10^2^	7.3811 × 10^4^	7.2417 × 10^1^	6.8586 × 10^1^	2.0427 × 10^3^	6.0964 × 10^1^	7.0062 × 10^1^
	Rank	6	9	5	8	4	3	7	2	1
	*p*-value	3.0199 × 10^−11^	3.0199 × 10^−11^	1.0105 × 10^−8^	3.0199 × 10^−11^	1.6351 × 10^−5^	2.3897 × 10^−8^	3.0199 × 10^−11^	3.0199 × 10^−11^	-
F12	Min	3.5279 × 10^6^	8.7017 × 10^10^	7.2668 × 10^6^	6.2728 × 10^10^	1.1258 × 10^7^	4.5434 × 10^7^	1.5888 × 10^9^	2.4553 × 10^10^	6.5751 × 10^5^
	Max	2.2512 × 10^10^	2.0672 × 10^11^	6.4227 × 10^7^	1.4081 × 10^11^	1.1308 × 10^8^	1.0011 × 10^9^	1.4224 × 10^10^	9.2162 × 10^10^	8.1294 × 10^6^
	Mean	6.7942 × 10^6^	1.5357 × 10^11^	2.7073 × 10^7^	1.0607 × 10^11^	5.6105 × 10^6^	2.7271 × 10^6^	5.1376 × 10^9^	5.3826 × 10^10^	4.2792 × 10^6^
	Std	1.4874 × 10^6^	2.9783 × 10^10^	1.3604 × 10^7^	2.1816 × 10^10^	3.3639 × 10^6^	2.5234 × 10^6^	2.6765 × 10^9^	1.5493 × 10^10^	1.7662 × 10^6^
	Rank	2	9	5	8	4	3	6	7	1
	*p*-value	3.0199 × 10^−11^	3.0199 × 10^−11^	4.0772 × 10^−11^	3.0199 × 10^−11^	2.1544 × 10^−10^	3.0199 × 10^−11^	3.0199 × 10^−11^	3.0199 × 10^−11^	-
F13	Min	7.2149 × 10^3^	4.9894 × 10^10^	5.9113 × 10^3^	2.5897 × 10^10^	4.6446 × 10^4^	2.7099 × 10^6^	1.1170 × 10^8^	2.0782 × 10^3^	2.1310 × 10^3^
	Max	6.0868 × 10^8^	1.4655 × 10^11^	3.8426 × 10^5^	1.0462 × 10^11^	4.0505 × 10^5^	1.4181 × 10^7^	1.1703 × 10^9^	3.8934 × 10^4^	3.0815 × 10^4^
	Mean	2.4268 × 10^4^	9.4367 × 10^10^	6.9790 × 10^4^	5.6101 × 10^10^	3.5581 × 10^4^	6.7661 × 10^6^	5.0369 × 10^8^	1.2327 × 10^4^	1.0811 × 10^4^
	Std	1.3717 × 10^4^	2.3674 × 10^10^	8.6118 × 10^4^	2.3213 × 10^10^	9.0619 × 10^3^	2.4739 × 10^6^	2.6966 × 10^8^	1.0564 × 10^4^	8.2004 × 10^3^
	Rank	5	9	4	8	3	6	7	2	1
	*p*-value	3.0199 × 10^−11^	3.0199 × 10^−11^	3.8202 × 10^−10^	3.0199 × 10^−11^	3.0199 × 10^−11^	3.0199 × 10^−11^	3.0199 × 10^−11^	3.0199 × 10^−11^	-
F14	Min	4.1906 × 10^5^	9.5570 × 10^7^	2.0714 × 10^3^	3.3316 × 10^7^	1.3498 × 10^5^	1.7340 × 10^4^	8.9321 × 10^5^	1.4359 × 10^4^	1.4389 × 10^4^
	Max	1.7106 × 10^7^	1.4629 × 10^9^	7.7314 × 10^4^	4.1784 × 10^8^	3.1740 × 10^6^	1.9287 × 10^6^	2.5760 × 10^7^	5.2479 × 10^5^	3.2952 × 10^5^
	Mean	1.0222 × 10^6^	4.6257 × 10^8^	1.7420 × 10^4^	1.9959 × 10^8^	1.2329 × 10^5^	8.7627 × 10^4^	7.0398 × 10^6^	6.2703 × 10^3^	1.2819 × 10^5^
	Std	2.9243 × 10^5^	3.3366 × 10^8^	1.7944 × 10^4^	1.1014 × 10^8^	8.1386 × 10^4^	8.2944 × 10^4^	5.3175 × 10^6^	5.0191 × 10^3^	8.5647 × 10^4^
	Rank	6	9	1	8	5	3	7	2	4
	*p*-value	3.0199 × 10^−11^	3.0199 × 10^−11^	2.8716 × 10^−10^	3.0199 × 10^−11^	1.5581 × 10^−8^	1.0035 × 10^−3^	3.0199 × 10^−11^	3.0199 × 10^−11^	-
F15	Min	1.3091 × 10^4^	1.0950 × 10^10^	3.1864 × 10^3^	7.8860 × 10^9^	9.5371 × 10^3^	1.0031 × 10^6^	2.2147 × 10^6^	2.1377 × 10^3^	1.7094 × 10^3^
	Max	6.7823 × 10^7^	6.4681 × 10^10^	1.8522 × 10^4^	3.5336 × 10^10^	1.0513 × 10^5^	2.6072 × 10^6^	7.3772 × 10^8^	2.0301 × 10^4^	2.0306 × 10^4^
	Mean	2.0082 × 10^4^	3.7251 × 10^10^	8.8352 × 10^3^	2.0862 × 10^10^	2.6560 × 10^4^	8.1477 × 10^3^	1.2709 × 10^8^	1.0394 × 10^4^	1.0118 × 10^4^
	Std	1.3254 × 10^7^	1.0897 × 10^10^	3.9229 × 10^3^	7.2317 × 10^9^	7.0281 × 10^3^	7.2640 × 10^3^	1.5770 × 10^8^	6.3665 × 10^3^	6.3766 × 10^3^
	Rank	6	9	1	8	5	4	7	3	2
	*p*-value	3.0199 × 10^−11^	3.0199 × 10^−11^	3.4783 × 10^−01^	3.0199 × 10^−11^	1.0937 × 10^−10^	3.0199 × 10^−11^	3.0199 × 10^−11^	3.0199 × 10^−11^	-
F16	Min	2.6894 × 10^3^	1.0329 × 10^4^	4.7435 × 10^3^	6.9585 × 10^3^	2.9053 × 10^3^	3.1301 × 10^3^	4.9183 × 10^3^	2.6587 × 10^3^	2.6772 × 10^3^
	Max	7.6050 × 10^3^	2.9300 × 10^4^	6.0319 × 10^3^	1.8262 × 10^4^	4.9114 × 10^3^	5.0726 × 10^3^	8.1525 × 10^3^	4.2215 × 10^3^	4.3269 × 10^3^
	Mean	7.0721 × 10^3^	1.6145 × 10^4^	5.4797 × 10^3^	1.2009 × 10^4^	3.6782 × 10^3^	4.1060 × 10^3^	6.3953 × 10^3^	3.5095 × 10^3^	3.3572 × 10^3^
	Std	1.8079 × 10^2^	4.0451 × 10^3^	3.2502 × 10^2^	2.8612 × 10^3^	3.0762 × 10^2^	3.5018 × 10^2^	8.3211 × 10^2^	4.3163 × 10^2^	4.5277 × 10^2^
	Rank	4	9	6	8	3	5	7	1	2
	*p*-value	3.0199 × 10^−11^	3.0199 × 10^−11^	3.0199 × 10^−11^	3.0199 × 10^−11^	9.5207 × 10^−4^	2.6015 × 10^−8^	3.0199 × 10^−11^	3.0199 × 10^−11^	-
F17	Min	2.4423 × 10^3^	9.8528 × 10^4^	3.6865 × 10^3^	3.3592 × 10^4^	2.6928 × 10^3^	2.9257 × 10^3^	3.3481 × 10^3^	2.4857 × 10^3^	2.2453 × 10^3^
	Max	6.2546 × 10^3^	9.5595 × 10^6^	4.4438 × 10^3^	2.9279 × 10^6^	4.2062 × 10^3^	4.1047 × 10^3^	6.2500 × 10^3^	4.1235 × 10^3^	3.6754 × 10^3^
	Mean	2.7730 × 10^3^	1.9614 × 10^6^	4.1175 × 10^3^	8.1293 × 10^5^	3.1007 × 10^3^	2.7561 × 10^3^	4.5374 × 10^3^	3.2171 × 10^3^	3.1866 × 10^3^
	Std	1.1315 × 10^2^	2.1509 × 10^6^	2.1428 × 10^2^	8.0943 × 10^5^	3.8834 × 10^2^	3.4257 × 10^2^	6.6839 × 10^2^	3.5632 × 10^2^	2.9674 × 10^2^
	Rank	2	9	6	8	5	3	7	4	1
	*p*-value	3.0199 × 10^−11^	3.0199 × 10^−11^	1.3289 × 10^−10^	3.0199 × 10^−11^	3.1830 × 10^−3^	7.6588 × 10^−5^	9.9186 × 10^−11^	3.0199 × 10^−11^	-
F18	Min	1.1442 × 10^6^	2.6311 × 10^8^	1.4109 × 10^5^	1.3777 × 10^8^	9.0091 × 10^5^	1.9368 × 10^5^	5.6824 × 10^6^	3.1599 × 10^5^	1.4013 × 10^5^
	Max	1.9841 × 10^8^	4.2097 × 10^9^	6.9597 × 10^6^	1.8157 × 10^9^	1.6783 × 10^7^	6.1907 × 10^6^	1.9516 × 10^8^	3.0602 × 10^6^	3.6657 × 10^6^
	Mean	2.2748 × 10^6^	1.6174 × 10^9^	1.0200 × 10^6^	5.1869 × 10^8^	6.5200 × 10^5^	1.4151 × 10^6^	7.8002 × 10^7^	6.2117 × 10^4^	1.2824 × 10^6^
	Std	9.3414 × 10^5^	9.0538 × 10^8^	1.2802 × 10^6^	3.8238 × 10^8^	3.7436 × 10^5^	1.5271 × 10^6^	5.7298 × 10^7^	3.1235 × 10^4^	9.1925 × 10^5^
	Rank	6	9	3	8	4	5	7	1	2
	*p*-value	3.0199 × 10^−11^	3.0199 × 10^−11^	7.2446 × 10^−2^	3.0199 × 10^−11^	4.4440 × 10^−7^	6.5671 × 10^−2^	3.0199 × 10^−11^	3.0199 × 10^−11^	-
F19	Min	2.2536 × 10^4^	7.2629 × 10^9^	2.7325 × 10^3^	1.7391 × 10^9^	4.9036 × 10^3^	4.3727 × 10^5^	1.5371 × 10^6^	2.7933 × 10^3^	2.2570 × 10^3^
	Max	1.0624 × 10^7^	2.5147 × 10^10^	3.3273 × 10^4^	1.3703 × 10^10^	5.3008 × 10^4^	8.5652 × 10^6^	2.0519 × 10^8^	4.3588 × 10^4^	4.3440 × 10^4^
	Mean	3.5934 × 10^4^	1.5889 × 10^10^	1.2450 × 10^4^	8.2284 × 10^9^	1.1180 × 10^4^	1.1636 × 10^4^	2.2289 × 10^7^	1.6594 × 10^4^	1.9110 × 10^4^
	Std	5.8193 × 10^3^	4.4925 × 10^9^	8.1019 × 10^3^	2.6890 × 10^9^	1.4291 × 10^4^	1.3499 × 10^4^	3.8275 × 10^7^	8.6516 × 10^3^	1.1465 × 10^4^
	Rank	5	9	1	8	4	6	7	3	2
	*p*-value	3.0199 × 10^−11^	3.0199 × 10^−11^	6.9724 × 10^−03^	3.0199 × 10^−11^	6.5671 × 10^−2^	3.0199 × 10^−11^	3.0199 × 10^−11^	3.0199 × 10^−11^	-
F20	Min	2.6998 × 10^3^	4.3422 × 10^3^	3.6976 × 10^3^	4.2261 × 10^3^	2.5538 × 10^3^	2.9170 × 10^3^	2.9470 × 10^3^	2.7100 × 10^3^	2.5200 × 10^3^
	Max	5.1469 × 10^0^	6.3104 × 10^3^	4.5766 × 10^3^	5.5410 × 10^3^	3.9869 × 10^3^	4.2865 × 10^3^	4.8530 × 10^3^	4.0657 × 10^3^	3.7274 × 10^3^
	Mean	2.8057 × 10^3^	5.5757 × 10^3^	4.2660 × 10^3^	5.0164 × 10^3^	2.9847 × 10^3^	2.7640 × 10^3^	3.9645 × 10^3^	3.2555 × 10^3^	3.1375 × 10^3^
	Std	1.1503 × 10^2^	3.9107 × 10^2^	1.8976 × 10^2^	2.8959 × 10^2^	2.6650 × 10^2^	3.5288 × 10^2^	4.5085 × 10^2^	2.8232 × 10^2^	2.8467 × 10^2^
	Rank	3	9	6	8	5	2	7	4	1
	*p*-value	3.0199 × 10^−11^	3.0199 × 10^−11^	3.3384 × 10^−11^	3.0199 × 10^−11^	1.2235 ×10^−1^	2.4327 × 10^−5^	4.1997 × 10^−10^	1.6132 × 10^−10^	-
F21	Min	2.3136 × 10^3^	3.4265 × 10^3^	2.7012 × 10^3^	3.1961 × 10^3^	2.4949 × 10^3^	2.6926 × 10^3^	2.8774 × 10^3^	2.4874 × 10^3^	2.4818 × 10^3^
	Max	3.0055 × 10^3^	3.8239 × 10^3^	2.7877 × 10^3^	3.7133 × 10^3^	2.7156 × 10^3^	2.9352 × 10^3^	3.3561 × 10^3^	2.7711 × 10^3^	2.6774 × 10^3^
	Mean	2.5208 × 10^3^	3.5781 × 10^3^	2.7456 × 10^3^	3.4235 × 10^3^	2.5004 × 10^3^	2.4284 × 10^3^	3.0989 × 10^3^	2.5924 × 10^3^	2.5879 × 10^3^
	Std	4.1582 × 10^1^	1.0084 × 10^2^	2.2641 × 10^1^	1.1081 × 10^2^	4.8130 × 10^1^	2.6054 × 10^1^	1.0630 × 10^2^	5.9981 × 10^1^	4.5289 × 10^1^
	Rank	2	9	5	8	3	4	7	6	1
	*p*-value	3.0199 × 10^−11^	3.0199 × 10^−11^	2.6099 × 10^−10^	3.0199 × 10^−11^	5.1060 × 10^−1^	6.0658 × 10^−11^	3.0199 × 10^−11^	3.0199 × 10^−11^	-
F22	Min	2.3290 × 10^3^	1.7731 × 10^4^	1.5921 × 10^4^	1.6734 × 10^4^	7.9563 × 10^3^	8.7394 × 10^3^	1.3148 × 10^4^	2.3027 × 10^3^	2.3083 × 10^3^
	Max	1.8295 × 10^4^	2.1117 × 10^4^	1.7677 × 10^4^	1.9531 × 10^4^	1.1998 × 10^4^	1.3645 × 10^4^	1.6183 × 10^4^	1.6584 × 10^4^	1.4202 × 10^4^
	Mean	7.2668 × 10^3^	1.9976 × 10^4^	1.6949 × 10^4^	1.8199 × 10^4^	8.5906 × 10^3^	8.6909 × 10^3^	1.5011 × 10^4^	9.7202 × 10^3^	1.0244 × 10^4^
	Std	1.7029 × 10^3^	7.5148 × 10^2^	4.1891 × 10^2^	6.5818 × 10^2^	8.8234 × 10^2^	1.5935 × 10^3^	7.7079 × 10^2^	1.6942 × 10^3^	1.4818 × 10^3^
	Rank	5	9	7	8	1	3	6	4	2
	*p*-value	3.0199 × 10^−11^	3.0199 × 10^−11^	3.3384 × 10^−11^	3.0199 × 10^−11^	8.7663 × 10^−1^	2.6806 × 10^−4^	1.6947 × 10^−9^	1.3289 × 10^−10^	-
F23	Min	2.9430 × 10^3^	4.3426 × 10^3^	3.1540 × 10^3^	4.3702 × 10^3^	2.9032 × 10^3^	3.7582 × 10^3^	3.2908 × 10^3^	2.8899 × 10^3^	2.8969 × 10^3^
	Max	3.5530 × 10^3^	6.1944 × 10^3^	3.2442 × 10^3^	5.3720 × 10^3^	3.1720 × 10^3^	5.1547 × 10^3^	4.3469 × 10^3^	3.2646 × 10^3^	3.2729 × 10^3^
	Mean	2.9640 × 10^3^	5.4133 × 10^3^	3.2063 × 10^3^	4.7249 × 10^3^	2.9374 × 10^3^	2.8589 × 10^3^	3.8112 × 10^3^	3.1530 × 10^3^	3.0477 × 10^3^
	Std	1.6073 × 10^1^	4.4709 × 10^2^	2.3231 × 10^1^	2.6141 × 10^2^	3.7229 × 10^1^	2.3081 × 10^1^	1.9865 × 10^2^	1.2025 × 10^2^	8.2490 × 10^1^
	Rank	2	9	5	8	1	6	7	3	4
	*p*-value	3.0199 × 10^−11^	3.0199 × 10^−11^	4.6159 × 10^−10^	3.0199 × 10^−11^	1.9073 × 10^−01^	3.0199 × 10^−11^	3.0199 × 10^−11^	3.0199 × 10^−11^	-
F24	Min	3.3535 × 10^3^	5.2235 × 10^3^	3.3085 × 10^3^	4.6051 × 10^3^	3.0156 × 10^3^	3.4696 × 10^3^	3.6496 × 10^3^	3.0843 × 10^3^	3.0889 × 10^3^
	Max	3.8130 × 10^3^	6.6783 × 10^3^	3.4137 × 10^3^	6.1114 × 10^3^	3.3977 × 10^3^	4.3417 × 10^3^	4.5317 × 10^3^	3.4981 × 10^3^	3.4223 × 10^3^
	Mean	3.3993 × 10^3^	6.0244 × 10^3^	3.3676 × 10^3^	5.1850 × 10^3^	3.0837 × 10^3^	3.1199 × 10^3^	3.9231 × 10^3^	3.3588 × 10^3^	3.2258 × 10^3^
	Std	4.3891 × 10^1^	3.7345 × 10^2^	3.3211 × 10^1^	3.7552 × 10^2^	3.3313 × 10^1^	1.2851 × 10^2^	1.8043 × 10^2^	1.2714 × 10^2^	8.7882 × 10^1^
	Rank	5	9	2	8	1	6	7	4	3
	*p*-value	3.0199 × 10^−11^	3.0199 × 10^−11^	5.9673 × 10^−9^	3.0199 × 10^−11^	4.3764 × 10^−1^	3.0199 × 10^−11^	3.0199 × 10^−11^	3.0199 × 10^−11^	-
F25	Min	2.9755 × 10^3^	4.7272 × 10^4^	3.1495 × 10^3^	2.8090 × 10^4^	3.0408 × 10^3^	2.9953 × 10^3^	4.0182 × 10^3^	3.0579 × 10^3^	3.0554 × 10^3^
	Max	1.4647 × 10^4^	9.0089 × 10^4^	3.3198 × 10^3^	6.2084 × 10^4^	3.1692 × 10^3^	3.1970 × 10^3^	6.4537 × 10^3^	3.1648 × 10^3^	3.1685 × 10^3^
	Mean	3.0165 × 10^3^	6.4324 × 10^4^	3.2248 × 10^3^	4.5618 × 10^4^	3.0272 × 10^3^	3.1182 × 10^3^	5.0816 × 10^3^	3.0624 × 10^3^	3.1108 × 10^3^
	Std	1.3439 × 10^1^	9.8094 × 10^3^	4.8319 × 10^1^	8.5251 × 10^3^	3.9176 × 10^1^	4.6437 × 10^1^	6.1735 × 10^2^	4.0919 × 10^1^	2.3322 × 10^1^
	Rank	1	9	6	8	2	5	7	4	3
	*p*-value	3.0199 × 10^−11^	3.0199 × 10^−11^	8.1527 × 10^−11^	3.0199 × 10^−11^	1.7613 × 10^−1^	1.3272 × 10^−2^	3.0199 × 10^−11^	3.0199 × 10^−11^	-
F26	Min	2.9444 × 10^3^	2.4665 × 10^4^	7.8425 × 10^3^	1.6339 × 10^4^	2.9634 × 10^3^	3.1709 × 10^3^	1.1662 × 10^4^	3.0121 × 10^3^	2.9194 × 10^3^
	Max	1.2607 × 10^4^	4.0057 × 10^4^	9.2694 × 10^3^	3.1395 × 10^4^	1.0315 × 10^4^	1.3575 × 10^4^	1.8656 × 10^4^	1.1977 × 10^4^	1.2784 × 10^4^
	Mean	4.7204 × 10^3^	3.2947 × 10^4^	8.4516 × 10^3^	2.6237 × 10^4^	5.6094 × 10^3^	5.2261 × 10^3^	1.4650 × 10^4^	7.1147 × 10^3^	8.8009 × 10^3^
	Std	1.5343 × 10^3^	4.6618 × 10^3^	3.2312 × 10^2^	3.5910 × 10^3^	8.4608 × 10^2^	3.5855 × 10^2^	1.5239 × 10^3^	3.9328 × 10^3^	3.0914 × 10^3^
	Rank	2	9	3	8	1	4	7	6	5
	*p*-value	2.6243 × 10^−3^	3.0199 × 10^−11^	1.3345 × 10^−1^	3.0199 × 10^−11^	9.5207 × 10^−4^	7.2951 × 10^−4^	3.0199 × 10^−11^	3.0199 × 10^−11^	-
F27	Min	3.3438 × 10^3^	7.1551 × 10^3^	3.6472 × 10^3^	6.2376 × 10^3^	3.3222 × 10^3^	4.0781 × 10^3^	4.0833 × 10^3^	3.3419 × 10^3^	3.3592 × 10^3^
	Max	3.2000 × 10^3^	1.1558 × 10^4^	4.1135 × 10^3^	9.2642 × 10^3^	3.6406 × 10^3^	6.8633 × 10^3^	6.2070 × 10^3^	3.9052 × 10^3^	4.0475 × 10^3^
	Mean	3.3665 × 10^3^	9.4605 × 10^3^	3.8592 × 10^3^	7.2641 × 10^3^	3.3638 × 10^3^	3.4086 × 10^3^	4.9736 × 10^3^	3.7284 × 10^3^	3.5766 × 10^3^
	Std	1.1946 × 10^1^	1.0557 × 10^3^	1.5081 × 10^2^	7.5261 × 10^2^	6.6761 × 10^1^	6.8591 × 10^1^	5.7337 × 10^2^	2.0288 × 10^2^	1.6445 × 10^2^
	Rank	2	9	6	8	1	5	7	3	4
	*p*-value	3.0199 × 10^−11^	3.0199 × 10^−11^	4.8011 × 10^−7^	3.0199 × 10^−11^	9.0000 × 10^−1^	4.0772 × 10^−11^	4.5043 × 10^−11^	3.0199 × 10^−11^	-
F28	Min	3.2686 × 10^3^	1.8715 × 10^4^	3.4037 × 10^3^	1.5041 × 10^4^	3.3066 × 10^3^	3.3134 × 10^3^	5.1531 × 10^3^	3.3186 × 10^3^	3.2791 × 10^3^
	Max	3.3000 × 10^3^	3.9762 × 10^4^	4.0746 × 10^3^	2.6058 × 10^4^	3.5665 × 10^3^	4.6174 × 10^3^	7.5274 × 10^3^	3.4944 × 10^3^	3.4946 × 10^3^
	Mean	3.2929 × 10^3^	2.5912 × 10^4^	3.6658 × 10^3^	2.0357 × 10^4^	3.3093 × 10^3^	3.3430 × 10^3^	5.9790 × 10^3^	3.2984 × 10^3^	3.3820 × 10^3^
	Std	1.2643 × 10^1^	4.5988 × 10^3^	1.3597 × 10^2^	3.0568 × 10^3^	2.2196 × 10^1^	4.2886 × 10^1^	5.3741 × 10^2^	3.4640 × 10^1^	4.3503 × 10^1^
	Rank	1	9	6	8	2	5	7	3	4
	*p*-value	5.5727 × 10^−10^	3.0199 × 10^−11^	3.3384 × 10^−11^	3.0199 × 10^−11^	4.2896 × 10^−1^	6.9522 × 10^−1^	3.0199 × 10^−11^	3.0199 × 10^−11^	-
F29	Min	3.8197 × 10^3^	1.2914 × 10^5^	5.1182 × 10^3^	2.7327 × 10^4^	4.4704 × 10^3^	5.0757 × 10^3^	6.9052 × 10^3^	3.4215 × 10^3^	3.6832 × 10^3^
	Max	1.4178 × 10^4^	9.3402 × 10^6^	6.3314 × 10^3^	7.8777 × 10^6^	5.9182 × 10^3^	8.1004 × 10^3^	1.4442 × 10^4^	5.4800 × 10^3^	5.3900 × 10^3^
	Mean	4.0094 × 10^3^	2.5219 × 10^6^	5.7297 × 10^3^	1.3381 × 10^6^	4.3452 × 10^3^	3.8221 × 10^3^	9.3579 × 10^3^	4.6241 × 10^3^	4.5194 × 10^3^
	Std	1.3811 × 10^2^	2.3693 × 10^6^	2.6551 × 10^2^	1.8461 × 10^6^	2.3220 × 10^2^	2.4027 × 10^2^	1.6141 × 10^3^	3.1504 × 10^2^	3.1315 × 10^2^
	Rank	4	9	6	8	5	3	7	2	1
	*p*-value	3.0199 × 10^−11^	3.0199 × 10^−11^	5.0723 × 10^−10^	3.0199 × 10^−11^	9.6263 × 10^−2^	3.0199 × 10^−11^	3.0199 × 10^−11^	3.0199 × 10^−11^	-
F30	Min	7.4175 × 10^5^	1.5664 × 10^10^	6.5073 × 10^6^	5.7609 × 10^9^	4.7658 × 10^6^	5.8641 × 10^7^	1.2543 × 10^8^	9.0351 × 10^8^	7.7096 × 10^5^
	Max	8.3117 × 10^8^	3.5848 × 10^10^	1.7274 × 10^7^	2.6506 × 10^10^	2.2023 × 10^7^	1.1202 × 10^8^	7.8791 × 10^8^	8.6897 × 10^9^	2.4072 × 10^6^
	Mean	8.4809 × 10^5^	2.3769 × 10^10^	1.1678 × 10^7^	1.2540 × 10^10^	1.6586 × 10^6^	2.4672 × 10^6^	3.4948 × 10^8^	3.5788 × 10^9^	1.2371 × 10^6^
	Std	5.8574 × 10^4^	5.7961 × 10^9^	2.6507 × 10^6^	5.2766 × 10^9^	3.0622 × 10^5^	6.2579 × 10^5^	1.6293 × 10^8^	1.8702 × 10^9^	3.9959 × 10^5^
	Rank	1	9	4	8	3	5	6	7	2
	*p*-value	3.0199 × 10^−11^	3.0199 × 10^−11^	3.0199 × 10^−11^	3.0199 × 10^−11^	3.0199 × 10^−11^	3.0199 × 10^−11^	3.0199 × 10^−11^	3.0199 × 10^−11^	-
+/=/−		30/0/0	30/0/0	27/0/3	30/0/0	18/0/12	26/0/4	30/0/0	30/0/0	-

#### 4.1.2. Convergence Behavior Analysis

**Figure 3:**
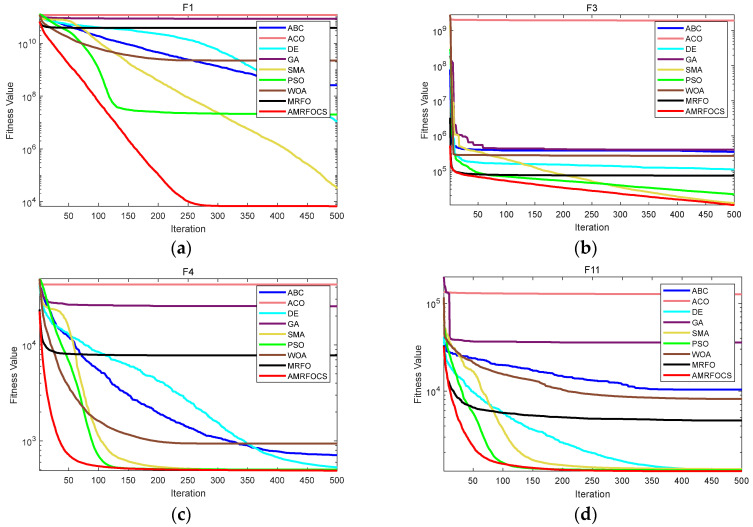

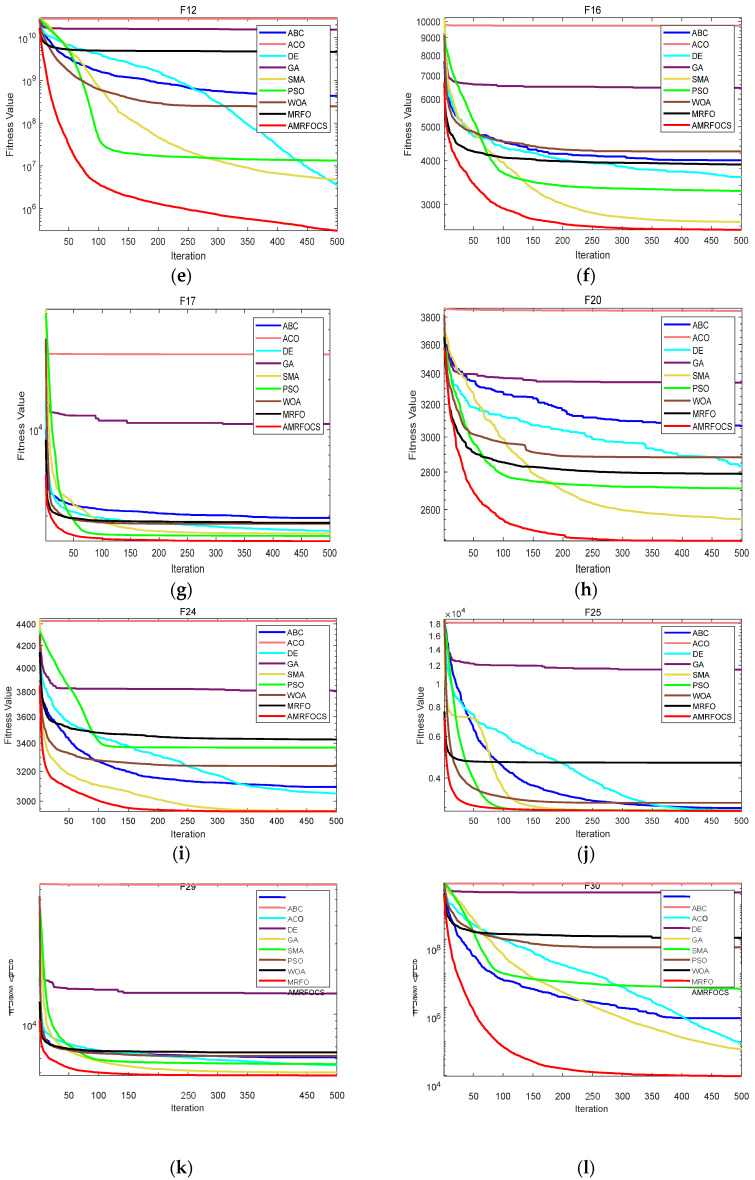
Mean convergence curves for AMRFOCS against other counterparts—CEC2017 benchmarks D = 30. (**a**) Convergence curve of F1. (**b**) Convergence curve of F3. (**c**) Convergence curve of F4. (**d**) Convergence curve of F11. (**e**) Convergence curve of F12. (**f**) Convergence curve of F16. (**g**) Convergence curve of F7. (**h**) Convergence curve of F20. (**i**) Convergence curve of F24. (**j**) Convergence curve of F25. (**k**) Convergence curve of F29. (**l**) Convergence curve of F30.

**Figure 4:**
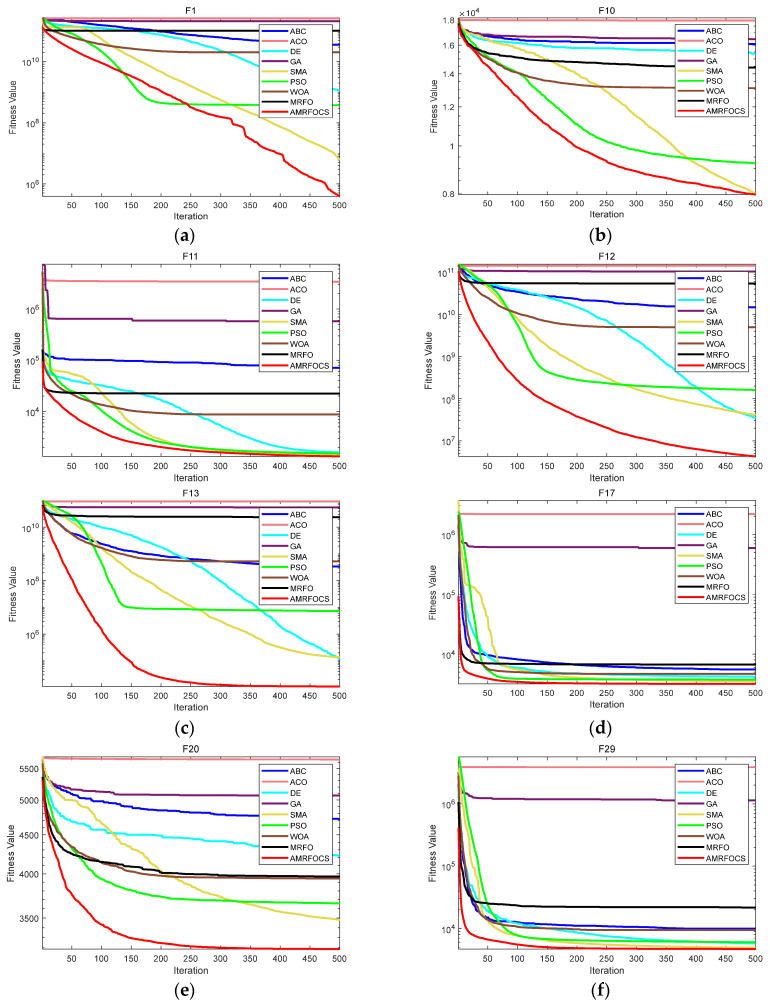
Mean convergence curves for AMRFOCS against other counterparts—CEC2017 benchmarks D = 50. (**a**) Convergence curve of F1. (**b**) Convergence curve of F10. (**c**) Convergence curve of F13. (**d**) Convergence curve of F17. (**e**) Convergence curve of F20. (**f**) Convergence curve of F29.

The second part is the convergence rate of the proposed AMRFOCS. Therefore, when dealing with 30-dimensional and 50-dimensional CEC2017 benchmark functions, the average convergence curve of AMRFOCS is compared with other classical algorithms, as shown in Figure 3 and Figure 4. By examining the curve, it can be recognized that many algorithms fall into the local solution of most functions. In the case of multimodal, mixed and composite functions such as F1, F3, F11, F12, F16, F17, F21, F24, F29 and F30, AMRFOCS shows a high degree of balance between the exploration and exploitation phases.

### 4.2. Valuation AMRFOCS by Utilizing CEC2020 Benchmark Functions

In this section, AMRFOCS passes 10 functional tests of CEC2020. According to the CEC2020 available dimension of the competition report, they are 5, 10, 15 and 20 [49]. Based on the experimental settings of the references, the dimensions used in this experiment are 10, 15 and 20 dimensions. The population sizes are 30, 100 and 30, respectively. The number of evaluation functions used is 3000, 500 and 5000, respectively. AMRFOCS is compared with ABC, ACO, SMA, GA, PSO, WOA and MRFO. Each algorithm implements 30 independent runs.

#### 4.2.1. Statistical Results Analysis

In this benchmark test of CEC2020, AMRFOCS is compared with seven algorithms, including ACO [42], ABC [43], GA, SMA [51], PSO [52], WOA [52] and MRFO [18].These comparative algorithms are from different references and show their respective advantages and good performance in the literature. The comparison results between AMRFOCS and the other algorithms are shown in Table 7, Table 8 and Table 9.

Table 7, Table 8 and Table 9 show four indicators, namely the mean value (mean), standard deviation (std), minimum value (min) and maximum value (max), as well as the sort value of the algorithm. In addition, at the 5% significance level, the Wilcoxon rank-sum test [47] is used to affirm whether AMRFOCS has a significant contribution to other algorithms. “-” represents ‘‘not applicable’’, which means that the best algorithm cannot be statistically compared with itself in the rank-sum test. In Table 7, Table 8 and Table 9, we give the algorithm’s ranking in different test functions and the *p*-value of the rank-sum test. The table’s bold data are the eight algorithms’ optimal minimum values (maximum values, mean values or standard deviation). Additionally, the last row of Table 7, Table 8 and Table 9 lists three symbols (+/−/=) to show the number of functions whereby AMRFOCS has a superior (+) performance, the number of functions whereby AMRFOCS has the same behavior as the other algorithms (=) and the number of functions whereby AMRFOCS is at a disadvantage.

Table 7, Table 8 and Table 9 show the average fitness values obtained for each method in the overall number of runs. Based on the results of these records, Table 7 shows that for both F1 and F2, AMRFOCS only achieved the best fitness value. In F3, AMRFOCS performed poorly, and PSO achieved the best mean and standard deviation. However, from F4 to F10, AMRFOCS showed a dominant position and basically achieved the best value of all indicators. Table 8 shows that of the 10 test functions in CEC2020, for 8 of them (F1, F4–F10), AMRFOCS performed better than all the other algorithms and showed the best average. As shown in Table 9, AMRFOCS still performed well in the reorganization experiment, especially with the mixed function and the combinatorial function. AMRFOCS always achieves the best fitness value and average value and ranks first.

The experimental results also show the standard deviation for AMRFOCS and the other comparison algorithms. Based on these results, AMRFOCS is the most stable metaheuristic algorithm because it enables more than half of the 10 functions to reach the minimum, such as F1, F4, F5, F7, F8, F9 and F10. In addition, the Wilcoxon signed-rank test is also shown in the table to check whether there is a statistical difference between AMRFOCS and the other algorithms when the *p* value is less than 0.05. The results show that most of the obtained values are less than 0.05, which means that there is a statistical difference between AMRFOCS and the other algorithms.

**Table 7:** Comparison of results obtained—CEC2020 benchmark functions (10D).

Function		ABC	ACO	SMA	GA	PSO	WOA	MRFO	AMRFOCS
F1	Min	1.1392 × 10^2^	1.2380 × 10^10^	4.1585 × 10^2^	4.1787 × 10^9^	1.0248 × 10^2^	5.2553 × 10^4^	1.0102 × 10^2^	1.0001 × 10^2^
	Max	3.7030 × 10^3^	4.0313 × 10^10^	1.2741 × 10^4^	2.7128 × 10^10^	5.5466 × 10^3^	2.2853 × 10^6^	6.8346 × 10^3^	8.8983 × 10^3^
	Mean	8.2827 × 10^2^	2.2807 × 10^10^	7.1025 × 10^3^	1.3071 × 10^10^	1.6719 × 10^3^	2.8538 × 10^5^	1.8831 × 10^3^	2.0195 × 10^3^
	Std	8.9945 × 10^2^	6.7663 × 10^9^	4.5802 × 10^3^	5.7495 × 10^9^	2.0858 × 10^3^	4.9750 × 10^5^	1.8434 × 10^3^	2.4139 × 10^3^
	Rank	1	8	5	7	2	6	3	4
	*p*-value	7.6183 × 10^−1^	3.0199 × 10^−11^	1.5292 × 10^−5^	3.0199 × 10^−11^	3.0199 × 10^−11^	3.0199 × 10^−11^	3.0199 × 10^−11^	-
F2	Min	2.1494 × 10^3^	2.7892 × 10^3^	1.2259 × 10^3^	2.6188 × 10^3^	1.2431 × 10^3^	1.4208 × 10^3^	1.2420 × 10^3^	1.1069 × 10^3^
	Max	2.7872 × 10^3^	4.6651 × 10^3^	2.0202 × 10^3^	3.5672 × 10^3^	2.3308 × 10^3^	2.5457 × 10^3^	2.5707 × 10^3^	2.1619 × 10^3^
	Mean	2.5096 × 10^3^	3.8503 × 10^3^	1.5427 × 10^3^	3.1849 × 10^3^	1.8046 × 10^3^	2.0166 × 10^3^	1.7194 × 10^3^	1.6420 × 10^3^
	Std	1.5423 × 10^2^	3.6014 × 10^2^	2.0360 × 10^2^	2.8795 × 10^2^	2.8154 × 10^2^	3.1524 × 10^2^	3.0358 × 10^2^	2.7249 × 10^2^
	Rank	5	8	1	7	4	3	6	2
	*p*-value	4.0772 × 10^−11^	3.0199 × 10^−11^	4.8413 × 10^−2^	3.0199 × 10^−11^	9.3341 × 10^−2^	5.1857 × 10^−7^	2.0283 × 10^−7^	-
F3	Min	7.2727 × 10^2^	1.0276 × 10^3^	7.1436 × 10^2^	8.9206 × 10^2^	7.1620 × 10^2^	7.4046 × 10^2^	7.1873 × 10^2^	7.1666 × 10^2^
	Max	7.4843 × 10^2^	1.4397 × 10^3^	7.4611 × 10^2^	1.1748 × 10^3^	7.2973 × 10^2^	8.1452 × 10^2^	7.8961 × 10^2^	7.7151 × 10^2^
	Mean	7.3954 × 10^2^	1.2033 × 10^3^	7.2509 × 10^2^	1.0282 × 10^3^	7.2131 × 10^2^	7.7289 × 10^2^	7.4756 × 10^2^	7.4002 × 10^2^
	Std	4.8970 × 10^0^	9.1232 × 10^1^	5.9102 × 10^0^	8.3489 × 10^1^	4.2931 × 10^0^	1.8023 × 10^1^	1.7360 × 10^1^	1.3309 × 10^1^
	Rank	3	8	2	7	1	5	6	4
	*p*-value	4.9178 × 10^−1^	3.0199 × 10^−11^	1.7479 × 10^−5^	3.0199 × 10^−11^	1.1536 × 10^−1^	4.1825 × 10^−9^	1.1023 × 10^−8^	-
F4	Min	1.9009 × 10^3^	3.9594 × 10^5^	1.9006 × 10^3^	1.4313 × 10^4^	1.9005 × 10^0^	1.9015 × 10^3^	1.9005 × 10^3^	1.9001 × 10^3^
	Max	1.9024 × 10^3^	2.1254 × 10^7^	1.9020 × 10^3^	3.9910 × 10^6^	1.9017 × 10^3^	1.9133 × 10^3^	1.9021 × 10^3^	1.9017 × 10^3^
	Mean	1.9018 × 10^3^	5.8465 × 10^6^	1.9010 × 10^3^	1.1194 × 10^6^	1.9010 × 10^3^	1.9052 × 10^3^	1.9011 × 10^3^	1.9010 × 10^3^
	Std	3.8056 × 10^−1^	5.3127 × 10^6^	3.6866 × 10^−1^	1.1438 × 10^6^	3.8581 × 10^−1^	3.0433 × 10^0^	4.0247 × 10^−1^	3.4371 × 10 ^-1^
	Rank	4	8	2	7	3	5	6	1
	*p*-value	2.6099 × 10^−10^	3.0199 × 10^−11^	2.6433 × 10^−1^	3.0199 × 10^−11^	2.0283 × 10^−7^	3.3384 × 10^−11^	3.0199 × 10^−11^	-
F5	Min	3.0234 × 10^4^	7.8639 × 10^5^	1.7319 × 10^3^	1.2076 × 10^5^	2.1097 × 10^3^	4.0071 × 10^3^	1.7394 × 10^3^	1.7216 × 10^3^
	Max	3.0470 × 10^5^	2.0144 × 10^8^	1.7902 × 10^4^	7.0618 × 10^7^	9.3423 × 10^3^	2.2519 × 10^6^	2.6867 × 10^3^	2.3247 × 10^3^
	Mean	9.7741 × 10^4^	3.5939 × 10^7^	7.4005 × 10^3^	1.0550 × 10^7^	4.5410 × 10^3^	3.2595 × 10^5^	2.1621 × 10^3^	2.0043 × 10^3^
	Std	7.7378 × 10^4^	4.2267 × 10^7^	5.9110 × 10^3^	1.7068 × 10^7^	2.6321 × 10^3^	5.8379 × 10^5^	2.6215 × 10^2^	1.7213 × 10^2^
	Rank	5	8	3	7	2	6	4	1
	*p*-value	3.0199 × 10^−11^	3.0199 × 10^−11^	2.7829 × 10^−07^	3.0199 × 10^−11^	6.5183 × 10^−09^	3.0199 × 10^−11^	1.0702 × 10^−9^	-
F6	Min	1.6004 × 10^3^	1.6107 × 10^3^	1.6002 × 10^3^	1.6035 × 10^3^	1.6007 × 10^3^	1.6126 × 10^3^	1.6000 × 10^3^	1.6000 × 10^3^
	Max	1.6010 × 10^3^	2.1694 × 10^3^	1.6010 × 10^3^	1.8803 × 10^3^	1.9365 × 10^3^	1.9815 × 10^3^	1.6012 × 10^3^	1.6006 × 10^3^
	Mean	1.6007 × 10^3^	1.7757 × 10^3^	1.6005 × 10^3^	1.7042 × 10^3^	1.7815 × 10^3^	1.8004 × 10^3^	1.6003 × 10^3^	1.6002 × 10^3^
	Std	1.6805 × 10^−1^	1.3259 × 10^2^	2.5080 × 10^−1^	7.1502 × 10^1^	8.9709 × 10^1^	1.0238 × 10^2^	2.9741 × 10^−1^	1.6704 × 10 ^-1^
	Rank	3	8	2	7	4	5	6	1
	*p*-value	1.5465 × 10^−9^	3.0199 × 10^−11^	8.5641 × 10^−4^	3.0199 × 10^−11^	8.1014 × 10^−10^	2.6015 × 10^−8^	2.1544 × 10^−10^	-
F7	Min	7.2813 × 10^3^	1.0613 × 10^4^	2.1235 × 10^3^	2.5605 × 10^4^	2.1008 × 10^3^	3.1679 × 10^3^	2.1006 × 10^3^	2.1001 × 10^3^
	Max	4.8656 × 10^4^	5.5590 × 10^7^	1.3274 × 10^4^	2.8168 × 10^7^	2.7098 × 10^3^	7.9900 × 10^4^	2.5875 × 10^3^	2.3049 × 10^3^
	Mean	2.1022 × 10^4^	1.3985 × 10^7^	4.2549 × 10^3^	3.9699 × 10^6^	2.3453 × 10^3^	2.1814 × 10^4^	2.2227 × 10^3^	2.1724 × 10^3^
	Std	1.0771 × 10^4^	1.4600 × 10^7^	3.2623 × 10^3^	6.1860 × 10^6^	1.7779 × 10^2^	1.8219 × 10^4^	1.2202 × 10^2^	6.8456 × 10^1^
	Rank	5	8	2	7	3	6	4	1
	*p*-value	3.0199 × 10^−11^	3.0199 × 10^−11^	9.0632 × 10^−8^	3.0199 × 10^−11^	3.0199 × 10^−11^	3.0199 × 10^−11^	4.9752 × 10^−11^	-
F8	Min	2.3042 × 10^3^	3.5079 × 10^3^	2.2226 × 10^3^	2.7068 × 10^3^	2.3006 × 10^3^	2.2835 × 10^3^	2.3003 × 10^3^	2.2181 × 10^3^
	Max	2.3090 × 10^3^	5.5522 × 10^3^	3.2109 × 10^3^	4.2787 × 10^3^	2.3042 × 10^3^	3.6330 × 10^3^	2.3062 × 10^3^	2.3032 × 10^3^
	Mean	2.3068 × 10^3^	4.5947 × 10^3^	2.4092 × 10^3^	3.4760 × 10^3^	2.3018 × 10^3^	2.4247 × 10^3^	2.3020 × 10^3^	2.2970 × 10^3^
	Std	1.3501 × 10^0^	5.6370 × 10^2^	2.5965 × 10^2^	4.6147 × 10^2^	8.2941 × 10^−1^	3.4389 × 10^2^	1.3591 × 10^0^	7.6795 × 10 ^-1^
	Rank	2	8	3	7	5	6	4	1
	*p*-value	3.0180 × 10^−11^	3.0180 × 10^−11^	2.9203 × 10^−2^	3.0180 × 10^−11^	3.0180 × 10^−11^	5.0695 × 10^−10^	3.0180 × 10^−11^	-
F9	Min	2.7173 × 10^3^	2.8592 × 10^3^	2.7433 × 10^3^	2.7979 × 10^3^	2.4000 × 10^3^	2.7527 × 10^3^	2.5000 × 10^3^	2.4000 × 10^3^
	Max	2.7698 × 10^3^	3.3082 × 10^3^	2.7753 × 10^3^	3.1470 × 10^3^	2.7677 × 10^3^	2.8258 × 10^3^	2.7668 × 10^3^	2.7597 × 10^3^
	Mean	2.7557 × 10^3^	3.0335 × 10^3^	2.7571 × 10^3^	2.9184 × 10^3^	2.6812 × 10^3^	2.7776 × 10^3^	2.6820 × 10^3^	2.6723 × 10^3^
	Std	1.2156 × 10^1^	1.0545 × 10^2^	9.2128 × 10^0^	7.6362 × 10^1^	1.2122 × 10^2^	2.0095 × 10^1^	1.1194 × 10^2^	8.3901 × 10^1^
	Rank	3	8	2	6	7	4	5	1
	*p*-value	1.2018 × 10^−8^	3.0180 × 10^−11^	6.7634 × 10^−5^	2.3701 × 10^−10^	2.1322 × 10^−5^	2.9201 × 10^−9^	6.9113 × 10^−4^	-
F10	Min	2.8979 × 10^3^	3.1575 × 10^3^	2.8982 × 10^3^	3.0391 × 10^3^	2.8979 × 10^3^	2.6443 × 10^3^	2.8979 × 10^3^	2.6000 × 10^3^
	Max	2.9460 × 10^3^	7.1006 × 10^3^	3.0242 × 10^3^	5.1923 × 10^3^	2.9500 × 10^3^	2.9723 × 10^3^	2.9489 × 10^3^	2.9495 × 10^3^
	Mean	2.9392 × 10^3^	4.8641 × 10^3^	2.9384 × 10^3^	3.8670 × 10^3^	2.9220 × 10^3^	2.9354 × 10^3^	2.9349 × 10^3^	2.9015 × 10^3^
	Std	9.7289 × 10^0^	8.7925 × 10^2^	2.8954 × 10^1^	5.8024 × 10^2^	2.3653 × 10^1^	7.1523 × 10^1^	1.9938 × 10^1^	2.2267 × 10^1^
	Rank	2	8	4	7	3	5	6	1
	*p*-value	5.7929 × 10^−1^	3.0199 × 10^−11^	1.3272 × 10^−2^	3.0199 × 10^−11^	9.3519 × 10^−1^	2.0152 × 10^−8^	4.5726 × 10^−9^	-
+/=/−		7/0/3	10/0/0	9/0/1	9/0/1	7/0/3	10/0/0	10/0/0	-

**Table 8:** Comparison of results obtained—CEC2020 benchmark functions (15D).

Function		ABC	ACO	SMA	GA	PSO [53]	WOA	MRFO	AMRFOCS
F1	Min	8.9182 × 10^3^	1.8080 × 10^10^	3.0812 × 10^2^	1.1723 × 10^10^	1.5726 × 10^6^	5.5228 × 10^7^	1.0003 × 10^2^	1.0919 × 10^2^
	Max	3.0062 × 10^6^	6.3113 × 10^10^	2.5958 × 10^4^	4.3013 × 10^10^	4.3153 × 10^6^	1.1845 × 10^9^	2.5394 × 10^4^	1.9760 × 10^4^
	Mean	2.0499 × 10^5^	3.9588 × 10^10^	1.1066 × 10^4^	2.4697 × 10^10^	2.8600 × 10^8^	3.5501 × 10^8^	8.3197 × 10^3^	6.2404 × 10^3^
	Std	5.7480 × 10^5^	1.0666 × 10^10^	9.1622 × 10^3^	7.0715 × 10^9^	6.5900 × 10^8^	2.6468 × 10^8^	8.0391 × 10^3^	5.8357 × 10^3^
	Rank	3	8	2	7	4	5	6	1
	*p*-value	1.6132 × 10^−10^	3.0199 × 10^−11^	1.3272 × 10^−2^	3.0199 × 10^−11^	3.0199 × 10^−11^	3.0199 × 10^−11^	3.0199 × 10^−11^	-
F2	Min	3.3808 × 10^3^	4.1367 × 10^3^	1.3889 × 10^3^	3.9989 × 10^3^	1.9509 × 10^3^	2.4380 × 10^3^	1.7015 × 10^3^	1.2364 × 10^3^
	Max	4.3815 × 10^3^	6.0406 × 10^3^	3.3464 × 10^3^	5.5303 × 10^3^	3.5246 × 10^3^	4.1739 × 10^3^	3.0513 × 10^3^	2.7292 × 10^3^
	Mean	4.0746 × 10^3^	5.4222 × 10^3^	2.0896 × 10^3^	4.7553 × 10^3^	3.9700 × 10^2^	3.3124 × 10^3^	2.2987 × 10^3^	1.9570 × 10^3^
	Std	2.6396 × 10^2^	4.1989 × 10^2^	4.0949 × 10^2^	4.0296 × 10^2^	1.9500 × 10^2^	4.7882 × 10^2^	3.4635 × 10^2^	3.6533 × 10^2^
	Rank	4	8	3	6	2	5	7	1
	*p*-value	3.0199 × 10^−11^	3.0199 × 10^−11^	1.3345 × 10^−1^	3.0199 × 10^−11^	2.0023 × 10^−6^	4.1997 × 10^−10^	9.9186 × 10^−11^	-
F3	Min	7.7125 × 10^2^	1.3751 × 10^3^	7.3112 × 10^2^	1.2423 × 10^3^	7.8084 × 10^2^	7.9357 × 10^2^	7.5401 × 10^2^	7.3690 × 10^2^
	Max	8.1444 × 10^2^	2.0440 × 10^3^	7.5919 × 10^2^	1.6998 × 10^3^	9.2097 × 10^2^	9.6361 × 10^2^	9.0212 × 10^2^	8.0621 × 10^2^
	Mean	7.9919 × 10^2^	1.7320 × 10^3^	7.4427 × 10^2^	1.4555 × 10^3^	2.2800 × 10^1^	8.7867 × 10^2^	8.0701 × 10^2^	7.6385 × 10^2^
	Std	9.3785 × 10^0^	1.7168 × 10^2^	8.4538 × 10^0^	1.2329 × 10^2^	3.3400 × 10^0^	4.3156 × 10^1^	3.6815 × 10^1^	1.8231 × 10^1^
	Rank	2	8	1	7	4	5	6	3
	*p*-value	8.5641 × 10^−4^	3.0199 × 10^−11^	1.4294 × 10^−8^	3.0199 × 10^−11^	4.9426 × 10^−5^	3.6897 × 10^−11^	4.5043 × 10^−11^	-
F4	Min	1.9062 × 10^3^	5.0856 × 10^5^	1.9012 × 10^3^	5.5274 × 10^4^	1.9048 × 10^3^	1.9173 × 10^3^	1.9015 × 10^3^	1.9009 × 10^3^
	Max	1.9101 × 10^3^	1.4279 × 10^7^	1.9053 × 10^3^	1.0775 × 10^7^	1.9093 × 10^3^	2.3598 × 10^3^	1.9102 × 10^3^	1.9044 × 10^3^
	Mean	1.9083 × 10^3^	5.7404 × 10^6^	1.9025 × 10^3^	2.6295 × 10^6^	7.5000 × 10^1^	2.0594 × 10^3^	1.9038 × 10^3^	1.9022 × 10^3^
	Std	8.4822 × 10^−1^	4.0652 × 10^6^	9.5245 × 10^−1^	2.3108 × 10^6^	4.0000 × 10^2^	1.1233 × 10^2^	1.8716 × 10^0^	9.6539 × 10^−1^
	Rank	3	8	4	7	2	5	6	1
	*p*-value	5.4941 × 10^−11^	3.0199 × 10^−11^	1.4128 × 10^−1^	3.0199 × 10^−11^	4.5043 × 10^−11^	3.0199 × 10^−11^	3.0199 × 10^−11^	-
F5	Min	9.8935 × 10^4^	8.4389 × 10^6^	3.3567 × 10^3^	6.2312 × 10^6^	1.7109 × 10^4^	9.1560 × 10^3^	2.7053 × 10^3^	1.8091 × 10^3^
	Max	1.4449 × 10^6^	3.6071 × 10^8^	1.0135 × 10^6^	2.9321 × 10^8^	3.9436 × 10^5^	3.0431 × 10^7^	1.7649 × 10^5^	4.9794 × 10^3^
	Mean	6.2043 × 10^5^	1.4181 × 10^8^	3.4353 × 10^5^	5.0984 × 10^7^	2.6300 × 10^4^	4.5353 × 10^6^	1.9206 × 10^4^	3.2198 × 10^3^
	Std	3.5873 × 10^5^	1.0360 × 10^8^	3.7205 × 10^5^	6.1107 × 10^7^	1.0100 × 10^5^	6.1643 × 10^6^	3.1836 × 10^4^	7.2998 × 10^2^
	Rank	4	8	3	7	2	6	5	1
	*p*-value	3.0199 × 10^−11^	3.0199 × 10^−11^	2.5721 × 10^−7^	3.0199 × 10^−11^	3.0199 × 10^−11^	3.0199 × 10^−11^	3.0199 × 10^−11^	-
F6	Min	1.7276 × 10^3^	1.7276 × 10^3^	1.7276 × 10^3^	1.7276 × 10^3^	1.7276 × 10^3^	1.7276 × 10^3^	1.7276 × 10^3^	1.6025 × 10^3^
	Max	1.7276 × 10^3^	1.7934 × 10^3^	1.7276 × 10^3^	1.7350 × 10^3^	1.7276 × 10^3^	1.7276 × 10^3^	1.7276 × 10^3^	1.6025 × 10^3^
	Mean	1.7276 × 10^3^	1.7359 × 10^3^	1.7276 × 10^3^	1.7285 × 10^3^	1.2800 × 10^2^	1.7276 × 10^3^	1.7276 × 10^3^	1.6025 × 10^3^
	Std	5.5011 × 10^−6^	1.4801 × 10^1^	1.0386 × 10^−8^	1.6969 × 10^0^	8.9900 × 10^1^	3.2909 × 10^−11^	3.2043 × 10^−7^	8.5918 × 10^−8^
	Rank	4	7	2	8	6	1	5	3
	*p*-value	7.6171 × 10^−3^	3.8461 × 10^−3^	2.5296 × 10^−4^	3.3384 × 10^−11^	2.0762 × 10^−6^	7.1086 × 10^−12^	6.2828 × 10^−6^	-
F7	Min	1.3476 × 10^5^	4.2064 × 10^6^	3.0138 × 10^3^	2.8154 × 10^6^	8.4201 × 10^3^	1.6493 × 10^5^	2.5435 × 10^3^	2.3344 × 10^3^
	Max	1.1855 × 10^6^	3.7604 × 10^08^	4.0915 × 10^5^	6.3958 × 10^7^	5.6134 × 10^5^	3.2858 × 10^7^	8.9311 × 10^3^	3.8247 × 10^3^
	Mean	5.9020 × 10^5^	5.8754 × 10^7^	1.4061 × 10^5^	2.4985 × 10^7^	4.9000 × 10^2^	8.3051 × 10^6^	4.3069 × 10^3^	2.7756 × 10^3^
	Std	3.2196 × 10^5^	6.6206 × 10^7^	1.3538 × 10^5^	1.7097 × 10^7^	4.1000 × 10^2^	8.8725 × 10^6^	1.5006 × 10^3^	3.7423 × 10^2^
	Rank	5	8	3	7	2	6	4	1
	h-value	1	1	1	1	1	1	1	-
	*p*-value	3.0199 × 10^−11^	3.0199 × 10^−11^	3.1589 × 10^−10^	3.0199 × 10^−11^	3.0199 × 10^−11^	3.0199 × 10^−11^	3.0199 × 10^−11^	-
F8	Min	2.5928 × 10^3^	4.9297 × 10^3^	2.3002 × 10^3^	2.7897 × 10^3^	2.3105 × 10^3^	2.3194 × 10^3^	2.2744 × 10^03^	2.2628 × 10^3^
	Max	5.5346 × 10^3^	7.4795 × 10^3^	3.7248 × 10^3^	6.6078 × 10^3^	4.5823 × 10^3^	5.1608 × 10^3^	2.3017 × 10^3^	2.3020 × 10^3^
	Mean	3.4110 × 10^3^	6.4724 × 10^3^	2.6974 × 10^3^	5.2093 × 10^3^	1.2300 × 10^2^	3.3445 × 10^3^	2.2998 × 10^3^	2.2983 × 10^3^
	Std	7.7193 × 10^2^	6.6794 × 10^2^	5.4439 × 10^2^	9.0966 × 10^2^	3.4200 × 10^1^	1.1929 × 10^3^	4.8385 × 10^0^	1.1649 × 10^0^
	Rank	4	8	2	7	3	6	5	1
	*p*-value	2.9710 × 10^−11^	2.9710 × 10^−11^	1.0531 × 10^−3^	2.9710 × 10^−11^	5.4938 × 10^−10^	2.9710 × 10^−11^	2.9710 × 10^−11^	-
F9	Min	2.8564 × 10^3^	3.2944 × 10^3^	2.7970 × 10^3^	3.0641 × 10^3^	2.5057 × 10^3^	2.8417 × 10^3^	2.5000 × 10^3^	2.7944 × 10^3^
	Max	2.8918 × 10^3^	4.4273 × 10^3^	2.8337 × 10^3^	3.8402 × 10^3^	3.2787 × 10^3^	3.0317 × 10^3^	2.8648 × 10^3^	2.8275 × 10^3^
	Mean	2.8760 × 10^3^	3.7546 × 10^3^	2.8108 × 10^3^	3.3587 × 10^3^	4.0600 × 10^2^	2.9344 × 10^3^	2.8016 × 10^3^	2.8082 × 10^3^
	Std	7.7323 × 10^0^	2.5499 × 10^2^	9.3661 × 10^0^	2.2100 × 10^2^	8.7800 × 10^1^	5.3179 × 10^1^	5.8581 × 10^1^	7.3602 × 10^0^
	Rank	3	8	1	7	6	4	5	2
	*p*-value	3.0199 × 10^−11^	3.0199 × 10^−11^	9.7052 × 10^−01^	3.0199 × 10^−11^	6.5277 × 10^−8^	4.6159 × 10^−10^	4.6159 × 10^−10^	-
F10	Min	2.9382 × 10^3^	5.7203 × 10^3^	2.9002 × 10^3^	4.3186 × 10^3^	2.9065 × 10^3^	3.1705 × 10^3^	2.9000 × 10^3^	2.9000 × 10^3^
	Max	3.1275 × 10^3^	1.9135 × 10^4^	3.1655 × 10^3^	1.2221 × 10^4^	3.1546 × 10^3^	3.5295 × 10^3^	3.1396 × 10^3^	3.1525 × 10^3^
	Mean	3.0845 × 10^3^	9.9196 × 10^3^	2.9642 × 10^3^	6.7857 × 10^3^	2.2100 × 10^4^	3.2974 × 10^3^	2.9296 × 10^3^	2.9175 × 10^3^
	Std	3.6737 × 10^1^	2.3530 × 10^3^	9.8522 × 10^1^	1.7801 × 10^3^	6.9600 × 10^1^	9.7382 × 10^1^	7.7440 × 10^1^	6.6683 × 10^1^
	Rank	3	8	4	7	1	5	6	2
	*p*-value	5.0650 × 10^−9^	9.4001 × 10^−12^	1.7736 × 10^−8^	9.4001 × 10^−12^	2.5168 × 10^−8^	2.4067 × 10^−11^	1.0445 × 10^−11^	-
+/=/−		10/0/0	10/0/0	7/0/3	10/0/0	10/0/0	10/0/0	10/0/0	-

**Table 9:** Comparison of results obtained—CEC2020 benchmark functions (20D).

Function		ABC	ACO	SMA [50]	GA	PSO [49,54]	WOA	MRFO [50,54]	AMRFOCS
F1	Min	6.6991 × 10^2^	4.0563 × 10^10^	1.6574 × 10^2^	2.5636 × 10^10^	1.0001 × 10^2^	6.3265 × 10^7^	8.3778 × 10^9^	1.0000 × 10^2^
	Max	1.2555 × 10^7^	8.4887 × 10^10^	1.2154 × 10^4^	6.7598 × 10^10^	3.3223 × 10^2^	6.1524 × 10^8^	3.7434 × 10^10^	5.8159 × 10^3^
	Mean	5.5245 × 10^5^	6.6290 × 10^10^	7.0214 × 10^3^	4.3889 × 10^10^	1.6732 × 10^2^	1.7326 × 10^8^	2.1385 × 10^10^	1.8646 × 10^3^
	Std	2.2918 × 10^6^	1.0292 × 10^10^	3.4661 × 10^3^	9.5270 × 10^9^	7.3804 × 10^1^	1.3456 × 10^8^	6.9336 × 10^9^	1.9147 × 10^3^
	Rank	4	8	3	7	2	5	6	1
	*p*-value	9.9410 × 10^−01^	3.0199 × 10^−11^	1.7290 × 10^−06^	3.0199 × 10^−11^	3.0199 × 10^−11^	3.0199 × 10^−11^	3.0199 × 10^−11^	-
F2	Min	5.1957 × 10^3^	5.8815 × 10^3^	1.1262 × 10^3^	5.0471 × 10^3^	1.1203 × 10^3^	3.1463 × 10^3^	1.7867 × 10^3^	1.7736 × 10^3^
	Max	5.9571 × 10^3^	7.7571 × 10^3^	2.0288 × 10^3^	6.8063 × 10^3^	2.0631 × 10^3^	5.0027 × 10^3^	3.6937 × 10^3^	3.3548 × 10^3^
	Mean	5.5760 × 10^3^	7.0582 × 10^3^	1.5103 × 10^3^	6.2467 × 10^3^	1.4822 × 10^3^	3.9622 × 10^3^	2.6240 × 10^3^	2.5471 × 10^3^
	Std	1.9190 × 10^2^	4.4158 × 10^2^	2.3456 × 10^2^	4.2793 × 10^2^	2.2322 × 10^02^	4.6842 × 10^2^	5.0980 × 10^2^	4.1138 × 10^2^
	Rank	6	8	1	7	4	3	5	2
	*p*-value	3.0199 × 10^−11^	3.0199 × 10^−11^	3.8249 × 10^−9^	3.0199 × 10^−11^	4.4205 × 10^−6^	5.4620 × 10^−6^	3.0199 × 10^−11^	-
F3	Min	8.2806 × 10^2^	1.8443 × 10^3^	7.2316 × 10^2^	1.4839 × 10^3^	7.0266 × 10^2^	8.4704 × 10^2^	7.5771 × 10^2^	7.6226 × 10^2^
	Max	8.7007 × 10^2^	2.6296 × 10^3^	7.3664 × 10^2^	2.4308 × 10^3^	7.3323 × 10^2^	1.0022 × 10^3^	9.2576 × 10^2^	9.3443 × 10^2^
	Mean	8.4879 × 10^2^	2.2196 × 10^3^	7.2929 × 10^2^	1.9061 × 10^3^	7.2179 × 10^2^	9.3951 × 10^2^	8.4981 × 10^2^	8.4617 × 10^2^
	Std	9.4200 × 10^0^	1.8780 × 10^2^	3.6765 × 10^0^	2.2344 × 10^2^	1.0121 × 10^1^	3.6276 × 10^1^	3.8601 × 10^1^	4.0698 × 10^1^
	Rank	3	8	2	7	1	5	6	4
	*p*-value	5.2014 × 10^−1^	3.0199 × 10^−11^	3.3384 × 10^−11^	3.0199 × 10^−11^	4.9426 × 10^−05^	2.0152 × 10^−08^	4.0772 × 10^−11^	-
F4	Min	1.9111 × 10^3^	1.2382 × 10^6^	1.9007 × 10^3^	4.3264 × 10^5^	1.9005 × 10^3^	1.9198 × 10^3^	1.9017 × 10^3^	1.9016 × 10^3^
	Max	1.9170 × 10^3^	2.2397 × 10^7^	1.9016 × 10^3^	1.2404 × 10^7^	1.9025 × 10^3^	2.6053 × 10^3^	1.9072 × 10^3^	1.9091 × 10^3^
	Mean	1.9136 × 10^3^	1.0108 × 10^7^	1.9012 × 10^3^	4.4157 × 10^6^	1.9015 × 10^3^	2.0567 × 10^3^	1.9043 × 10^3^	1.9046 × 10^3^
	Std	1.5087 × 10^0^	6.3959 × 10^6^	2.3298 × 10^0^	3.1502 × 10^6^	4.0505 ×10^−1^	1.8873 × 10^2^	1.2803 × 10^0^	2.0092 × 10^0^
	Rank	4	8	2	7	1	5	6	3
	*p*-value	2.9215 × 10^−9^	3.0199 × 10^−11^	1.1077 × 10^−06^	3.0199 × 10^−11^	2.7829 × 10^−07^	4.0772 × 10^−11^	3.0199 × 10^−11^	-
F5	Min	8.0766 × 10^5^	1.7852 × 10^7^	3.7210 × 10^3^	4.4310 × 10^6^	2.6626 × 10^3^	2.0301 × 10^5^	1.8919 × 10^3^	2.8351 × 10^3^
	Max	5.1985 × 10^6^	2.6166 × 10^8^	1.6802 × 10^4^	2.4465 × 10^8^	1.4404 × 10^5^	5.2724 × 10^6^	3.0040 × 10^3^	1.8735 × 10^4^
	Mean	2.4414 × 10^6^	1.3021 × 10^8^	1.3273 × 10^4^	6.2227 × 10^7^	1.2921 × 10^4^	2.2840 × 10^6^	2.4118 × 10^3^	6.1639 × 10^3^
	Std	9.3740 × 10^5^	6.5744 × 10^7^	4.0755 × 10^3^	4.8280 × 10^7^	2.5004 × 10^4^	1.5022 × 10^6^	2.9496 × 10^2^	4.1052 × 10^3^
	Rank	5	8	3	7	2	4	6	1
	*p*-value	3.0199 × 10^−11^	3.0199 × 10^−11^	3.3384 × 10^−11^	3.0199 × 10^−11^	9.9186 × 10^−11^	3.0199 × 10^−11^	3.0199 × 10^−11^	-
F6	Min	2.0209 × 10^3^	2.0209 × 10^3^	1.6025 × 10^3^	2.0209 × 10^3^	1.6025 × 10^3^	2.0209 × 10^3^	1.6025 × 10^3^	1.6622 × 10^3^
	Max	2.0209 × 10^3^	2.0209 × 10^3^	2.3267 × 10^3^	2.0209 × 10^3^	2.3267 × 10^3^	2.0209 × 10^3^	2.3267 × 10^3^	1.6622 × 10^3^
	Mean	2.0209 × 10^3^	2.0209 × 10^3^	1.8192 × 10^3^	2.0209 × 10^3^	1.8192 × 10^3^	2.0209 × 10^3^	1.8192 × 10^3^	1.6622 × 10^3^
	Std	2.3126 × 10^−13^	2.3126 × 10^−13^	2.1374 × 10^2^	2.3126 × 10^−13^	2.1374 × 10^2^	2.3126 × 10^−13^	2.1374 × 10^2^	2.3126 × 10^-13^
	Rank	5	6	2	7	3	8	4	1
	*p*-value	NaN	NaN	NaN	NaN	NaN	NaN	NaN	-
F7	Min	2.2983 × 10^5^	7.5943 × 10^6^	2.3186 × 10^3^	1.9998 × 10^6^	2.1388 × 10^3^	4.2518 × 10^4^	2.1809 × 10^3^	2.3988 × 10^3^
	Max	1.5042 × 10^6^	6.5444 × 10^8^	6.4946 × 10^3^	8.0646 × 10^7^	3.8441 × 10^3^	2.8797 × 10^6^	3.2587 × 10^3^	5.8574 × 10^3^
	Mean	7.9336 × 10^5^	1.2540 × 10^8^	3.8725 × 10^3^	2.7422 × 10^7^	2.5657 × 10^3^	8.9956 × 10^5^	2.6459 × 10^3^	3.7674 × 10^3^
	Std	3.4751 × 10^5^	1.2646 × 10^8^	1.3768 × 10^3^	2.3393 × 10^7^	3.5521 × 10^2^	8.6057 × 10^5^	2.4382 × 10^2^	1.0698 × 10^3^
	Rank	6	8	3	7	1	4	5	2
	*p*-value	3.0199 × 10^−11^	3.0199 × 10^−11^	3.3384 × 10^−11^	3.0199 × 10^−11^	2.1544 × 10^−10^	3.0199 × 10^−11^	3.0199 × 10^−11^	-
F8	Min	4.1639 × 10^3^	6.3323 × 10^3^	2.3000 × 10^3^	5.1673 × 10^3^	2.3000 × 10^03^	2.3252 × 10^3^	2.3000 × 10^3^	2.3000 × 10^3^
	Max	7.4706 × 10^3^	9.6924 × 10^3^	4.4908 × 10^3^	8.6986 × 10^3^	3.8809 × 10^03^	7.0329 × 10^3^	2.3028 × 10^3^	2.3025 × 10^3^
	Mean	6.6378 × 10^3^	8.6618 × 10^3^	3.9746 × 10^3^	7.4038 × 10^3^	2.9396 × 10^03^	4.4362 × 10^3^	2.3011 × 10^3^	2.3007 × 10^3^
	Std	9.3263 × 10^2^	7.3792 × 10^2^	4.3257 × 10^2^	7.4140 × 10^2^	4.8483 × 10^02^	1.8870 × 10^3^	6.9236 × 10^−1^	7.8838 × 10 ^-1^
	Rank	6	8	2	7	3	4	5	1
	*p*-value	2.6203 × 10^−11^	2.6203 × 10^−11^	3.8409 × 10^−06^	2.6203 × 10^−11^	2.6203 × 10^−11^	2.6203 × 10^−11^	2.6203 × 10^−11^	-
F9	Min	2.9048 × 10^3^	3.3682 × 10^3^	2.8289 × 10^3^	3.2094 × 10^3^	2.8125 × 10^3^	2.8789 × 10^3^	2.4379 × 10^3^	2.5000 × 10^3^
	Max	2.9581 × 10^3^	4.2698 × 10^3^	2.8727 × 10^3^	3.8063 × 10^3^	2.8470 × 10^3^	3.1286 × 10^3^	3.0148 × 10^3^	2.9151 × 10^3^
	Mean	2.9416 × 10^3^	3.8519 × 10^3^	2.8507 × 10^3^	3.4810 × 10^3^	2.8207 × 10^3^	3.0257 × 10^3^	2.9098 × 10^3^	2.8505 × 10^3^
	Std	1.0805 × 10^1^	2.3544 × 10^2^	1.2047 × 10^1^	1.6779 × 10^2^	9.6248 × 10^0^	5.2418 × 10^1^	1.1079 × 10^2^	2.1817 × 10^1^
	Rank	3	8	1	7	5	4	6	2
	*p*-value	3.6897 × 10^−11^	3.0199 × 10^−11^	1.8577 × 10^−1^	3.0199 × 10^−11^	3.0199 × 10^−11^	6.0658 × 10^−11^	3.0199 × 10^−11^	-
F10	Min	2.9067 × 10^3^	6.2467 × 10^3^	2.9100 × 10^3^	5.2774 × 10^3^	2.8992 × 10^3^	2.9798 × 10^3^	2.9114 × 10^3^	2.8997 × 10^3^
	Max	3.0033 × 10^3^	2.4861 × 10^4^	2.9139 × 10^3^	1.6018 × 10^4^	2.9605 × 10^3^	3.2326 × 10^3^	3.0078 × 10^3^	3.0024 × 10^3^
	Mean	2.9211 × 10^3^	1.4171 × 10^4^	2.9132 × 10^3^	8.7877 × 10^3^	2.9193 × 10^3^	3.0608 × 10^3^	2.9654 × 10^3^	2.9570 × 10^3^
	Std	2.6124 × 10^1^	4.2631 × 10^3^	1.4034 × 10^0^	2.9286 × 10^3^	1.3624 × 10^1^	4.9901 × 10^1^	3.2780 × 10^1^	3.1937 × 10^1^
	Rank	1	8	2	7	3	5	6	4
	*p*-value	5.5727 × 10^−10^	3.0199 × 10^−11^	1.1567 × 10^−07^	3.0199 × 10^−11^	2.7086 × 10^−02^	4.1127 × 10^−07^	3.0199 × 10^−11^	-
+/=/−		7/0/3	9/0/1	8/0/2	9/0/1	9/0/1	9/0/1	9/0/1	-

#### 4.2.2. Convergence Behavior Analysis

**Figure 5:**
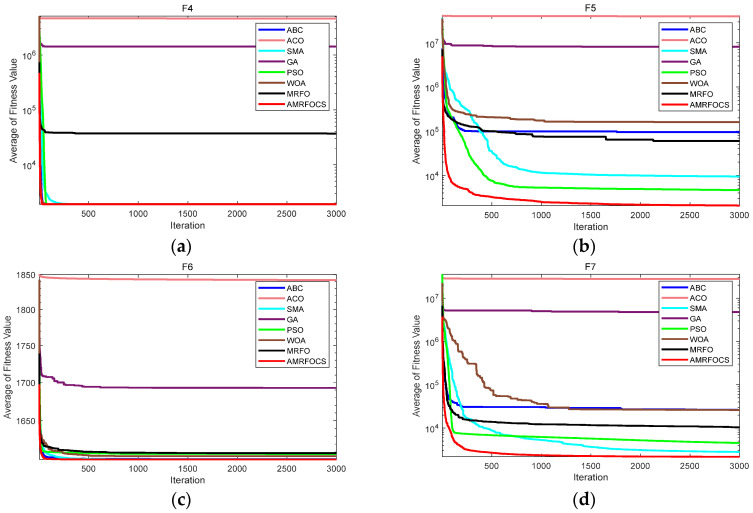

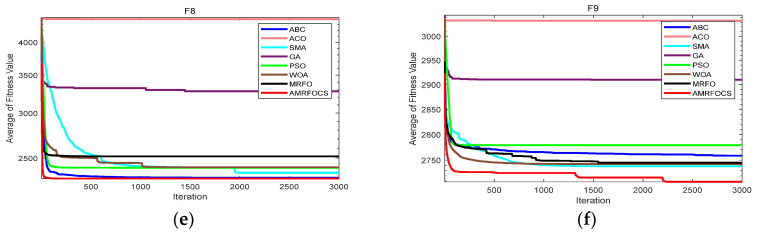
Mean convergence curves for AMRFOCS against other counterparts—CEC2020 benchmarks D = 10. (**a**) Convergence curve of F4. (**b**) Convergence curve of F5. (**c**) Convergence curve of F6. (**d**) Convergence curve of F7. (**e**) Convergence curve of F8. (**f**) Convergence curve of F9.

**Figure 6:**
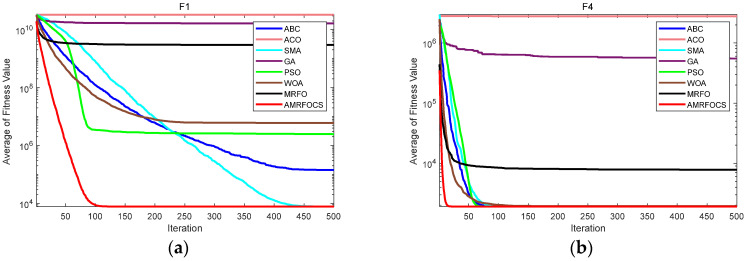

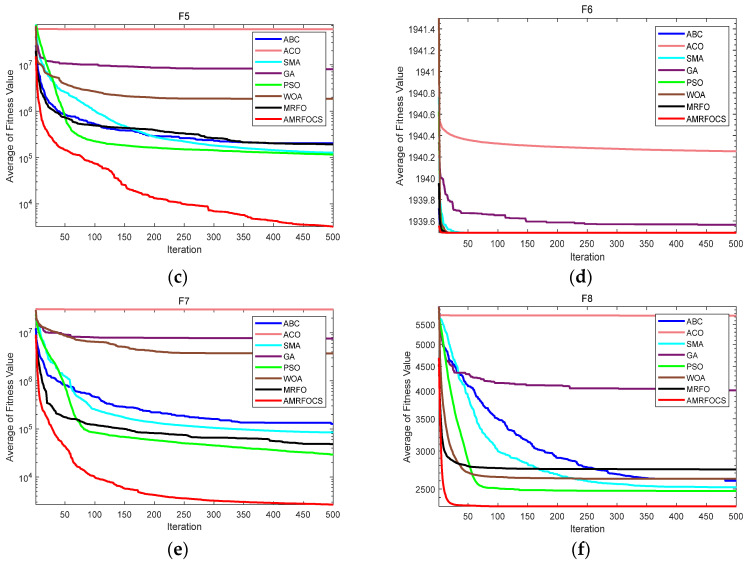

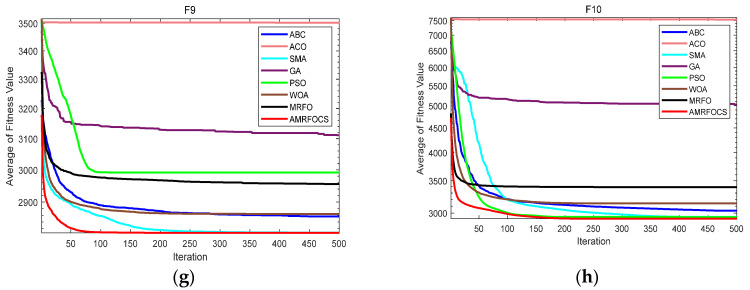
Mean convergence curves for AMRFOCS against other counterparts—CEC2020 benchmarks D = 15. (**a**) Convergence curve of F1. (**b**) Convergence curve of F4. (**c**) Convergence curve of F5. (**d**) Convergence curve of F6. (**e**) Convergence curve of F7. (**f**) Convergence curve of F8. (**g**) Convergence curve of F9. (**h**) Convergence curve of F10.

**Figure 7:**
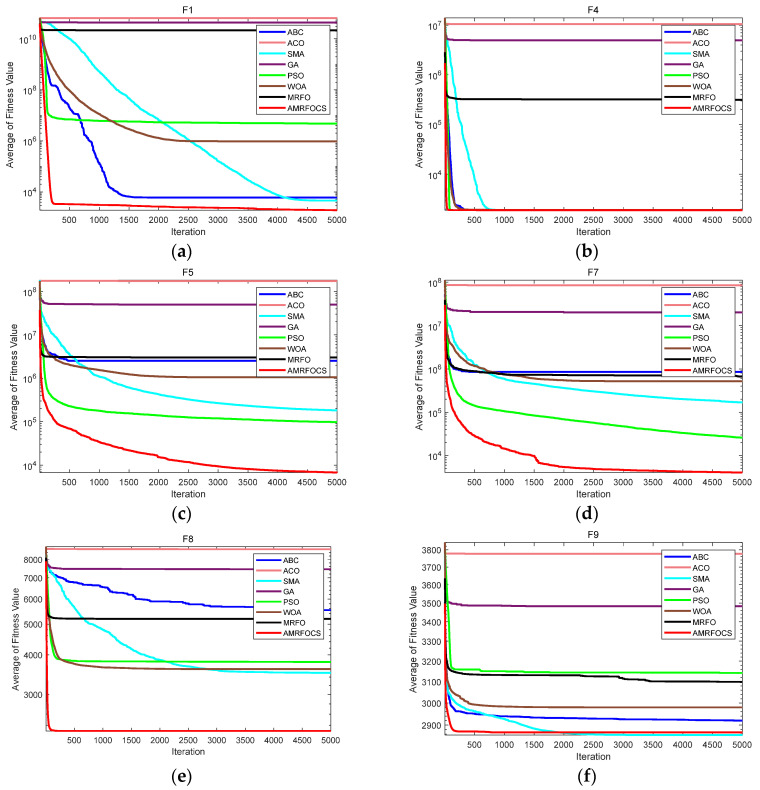
Mean convergence curves for AMRFOCS against other counterparts—CEC2020 benchmarks D = 20. (**a**) Convergence curve of F1. (**b**) Convergence curve of F4. (**c**) Convergence curve of F5. (**d**) Convergence curve of F7. (**e**) Convergence curve of F8. (**f**) Convergence curve of F9.

In order to further analyze the recommended AMRFOCS, the representations of the convergence curves of AMRFOCS and the other kinds of algorithms are plotted in Figure 5, Figure 6 and Figure 7 to assess the performance of AMRFOCS with the CEC2020 function. These results show that AMRFOCS is capable of faster convergence than all the compared algorithms, especially those in F5, F7, F8, F9 and F10. In different dimensions, the comprehensive performance of AMRFOCS ranks first. In more than 50% of the test functions, it can always reach the optimal value in less time and fewer iteration times.

## 5. AMRFOCS for Wireless Sensor Network (WSN)

In terms of deploying wireless sensor networks on 3D surfaces [55], compared with other deployment strategies, swarm intelligence optimization algorithms have the advantages of simplicity, ease of use and no need for special modeling [56]. In this paper, a new hybrid algorithm based on MRFO is proposed and applied to the deployment of a 3D-surface WSN. In the background of a complex environment, fixed simple and complex surfaces are set in the experiment [57].

### 5.1. WSN Coverage Model

In WSNs, the sensing radius *R* and communication radius *R_c_* of sensor nodes are fixed as *R_c_* = 2*R*. When deploying *N* homogeneous sensors in this area, the node set can be expressed as *Z* = {*z*_1_, *z*_2_, *z*_3_, …, *z_n_*}, which has the same sensing radius *R* and communication radius *R_c_*. In this paper, the Boolean model is used as the node perception model and grid segmentation method. As long as the target is within the node perception range, it can be successfully perceived. Assuming that the coordinates of a monitored node *z_i_* are (*x_i_*, *y_i_*, *z_i_*) and the position coordinates of the target point *d_j_* are (*x_i_*, *y_i_*, *z_i_*), the distance between the node and the target point is

(11) $$d(z_{i},d_{j})=\sqrt{{\left(x_{i}-y_{j}\right)}^{2}+{\left(y_{i}-y_{j}\right)}^{2}+{\left(z_{i}-z_{j}\right)}^{2}}$$

where *P*(*z_i_*, *d_j_*) represents the perceived quality of the node to the target node, as shown in (12). When the sensing probability is less than one, in order to improve the sensing quality of the target, multiple sensors are required to detect the target cooperatively, as shown in (13):

(12) $$P(z_{i},d_{j})=\left\{ \begin{array}{ll} 1 & \;\;\;\;\;\;d(z_{i},d_{j})\leq R\;\\ 0 & \;\;\;\;\;\;otherwise \end{array}\right.$$

(13) $$P(z_{i},d_{j})=1-\prod_{i=1}^{N}\left[1-P(z_{i},d_{j})\right]$$

The coverage of the monitoring area is the ratio of the number of target points covered by all sensor nodes to the total number of target points in the area, which is defined as

(14) $$S_{\mathrm{cov}}=\frac{\sum_{x=1}^{L}\sum_{y=1}^{W}P(z_{i},d_{j})\times SA_{(x-1)\cdot W+y}}{SA_{a}}$$

Taking Equation (14) as the objective function to obtain the maximum value is optimal. 

### 5.2. WSN Deployment on 3D Surface

Compared with the simple s in WSNs, the sensing radius *R* and communication radius *R_c_* of the sensor nodes are kept as *R_c_* = 2*R*. When deploying *N* homogeneous sensors in this area, the node set can be expressed as *Z* = {*z*_1_, *z*_2_, *z*_3_, …, *z_n_*}, which has the same sensing radius and communication radius *R_c_*. In this paper, the Boolean model is used as the node perception model and grid segmentation method. As long as the target is within the node perception range, it can be successfully perceived. Assuming that the coordinates of the monitored node *z_i_* are (*x_i_*, *y_i_*, *z_i_*) and the position coordinates of the target point *d_j_* are (*x_i_*, *y_i_*, *z_i_*), the distance between the node and the target point can be determined.

**Figure 8:**
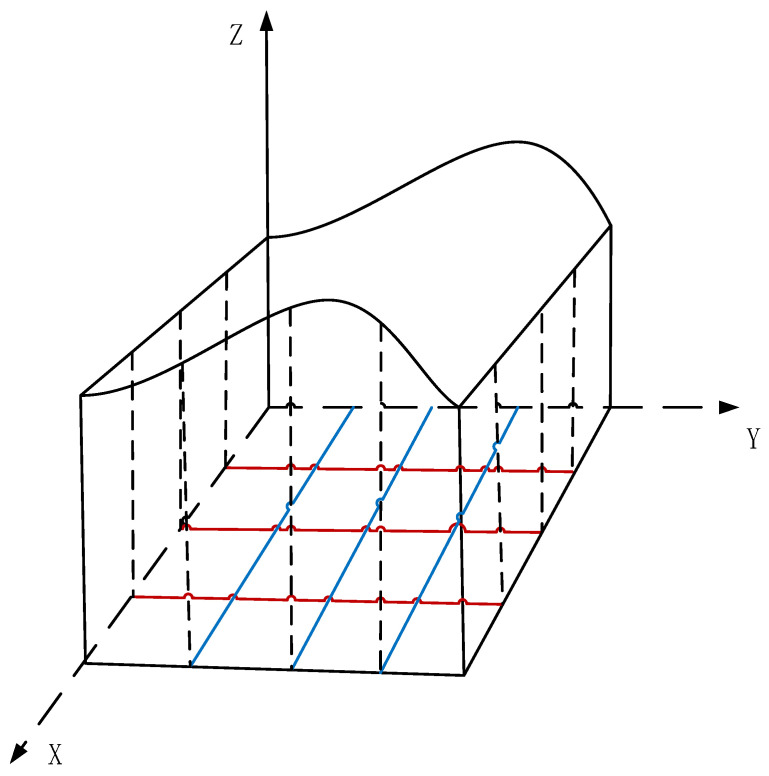
The schematic diagram of grid method (3D surface is vertically mapped to 2D plane).

Compared with the calculation of the surface area on the two-dimensional plane, the three-dimensional (3D) surface deployment is more complex and worthy of discussion. First, the three-dimensional undulating surface is not easy to divide equally. In order to solve this problem, a grid method is proposed (as shown in Figure 8): the 3D surface is vertically mapped to the 2D plane and the area is equally divided into a small grid, and the surface area is calculated by the approximate mapping between the two.

Equation (15) represents the surface equation, and the total area is obtained by dividing the curved area according to Equation (16):

(15) z=f(x,y)

(16) $$SA=\iint_{D_{xy}}\sqrt{1+{\left(\frac{dz}{dx}\right)}^{2}+{\left(\frac{dz}{dy}\right)}^{2}}dxdy$$

This experiment assumes that the energy consumption of nodes is not considered when deploying nodes, which only needs to optimize the coverage area of the network. The more area the network covers, the more properly the nodes are developed. Therefore, the goal of this experiment is to find the maximum coverage.

**Figure 9:**
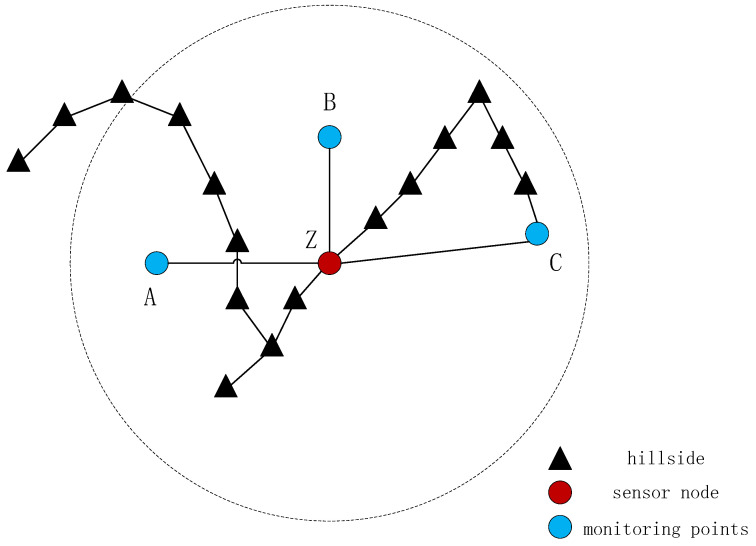
The schematic diagram of sensing blind area in the process of deploying nodes.

The Boolean perception model cannot intuitively describe the whole process of deploying sensor networks on 3D surfaces. As shown in Figure 9, the solid curve represents the surface, and the dotted circle determines the sensing boundary. The monitoring points shown in the figure are all within the sensing range of node *Z*. The signal transmitted in a straight line will be blocked by the nearby hillside, resulting in the interruption of the sensing signal, which is generally called the perception blind spot. Mathematically, the position relationship is checked according to the number of intersection points between the linear equation and the surface equation. For example, the intersection points between the spatial line segment and the surface between the monitoring point *B* and the sensor node *Z* are only two, *B* and *Z*, indicating that *B* can be completely covered by *Z*. Then, the equations can be established to obtain the number of solutions. The simultaneous equations are as follows: Equation (17).

(17) $$\left\{ \begin{array}{l} \frac{x-x_{1}}{x_{2}-x_{1}}=\frac{y-y_{1}}{y_{2}-y_{1}}=\frac{z-z_{1}}{z_{2}-z_{1}}\\ z=f(x,y) \end{array}\right.$$

In the instance of *Z* and *C* in the graph, the condition of coverage also needs to add a constraint. The midpoint of the line segment is in the inferior part of curve, which means that the monitoring points cannot be covered. The equation is shown in Equation (18):

(18) $$P(S,D)=\left\{ \begin{array}{ll} 1 & \;\;\;\;\;\;\;\;\;\;\;\;if(x_{3},y_{3})\leq z_{3}\\ 0 & \;\;\;\;\;\;\;\;\;\;\;\mathrm{otherwise} \end{array}\right.$$

When deploying wireless sensors, network connectivity is also one of the basic requirements. In this experiment, in order to facilitate the calculation, the communication radius is set to be twice the sensing radius. As shown in the figure, the Euclidean distance between *Z* and *A* is less than the communication radius, and there is a slope blocking the signal between *Z* and *A*, so *Z* and *A* cannot communicate with each other. This phenomenon is similar to the perceptual blind spot.

### 5.3. Results and Discussion

In order to verify the applicability and reliability of the AMRFOCS algorithm when deploying 3D wireless sensor networks, two 3D surfaces with different complexities are deployed. At the same time, they are compared with four 3D deployment methods optimized by metaheuristic algorithms, namely MRFO in [18], GWO in [58], WOA in [16] and PSO in [59]. Considering the fairness of the comparison, the parameter setting refers to the literature in the same field: the population size is 50 and the maximum number of iterations is 300.

First, a simple saddle-shaped surface is shown in Equation (19). The curved surface is segmented according to the grid method in Section 5.2, and the number of nodes is set to 50. Secondly, the complex surface is shown in Equation (20). The sensing radius of the node is 1 m, and the communication radius is 2 m. Due to the increase in the surface complexity, the sensor node of the experiment is boosted to 100:

(19) $$z=1-x^{2}-y^{2}$$

(20) z=sin(x)sin(y)

**Figure 10:**
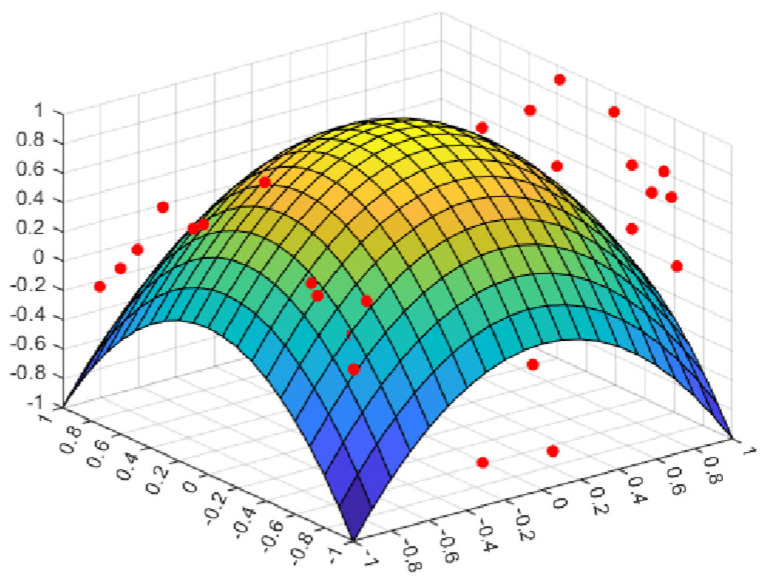
Initial nodes deployment diagram for a simple saddle-shaped surface.

**Figure 11:**
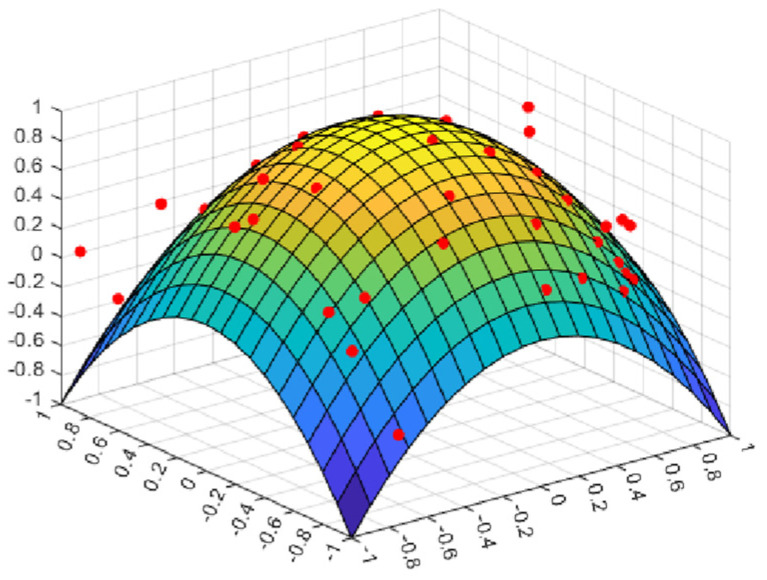
Optimized nodes deployment diagram for a simple saddle-shaped surface.

**Figure 12:**
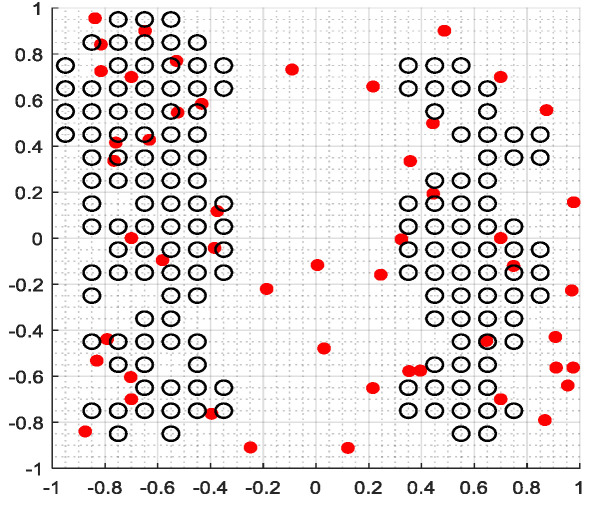
2D viewgraph of initial deployment for a simple saddle-shaped surface.

**Figure 13:**
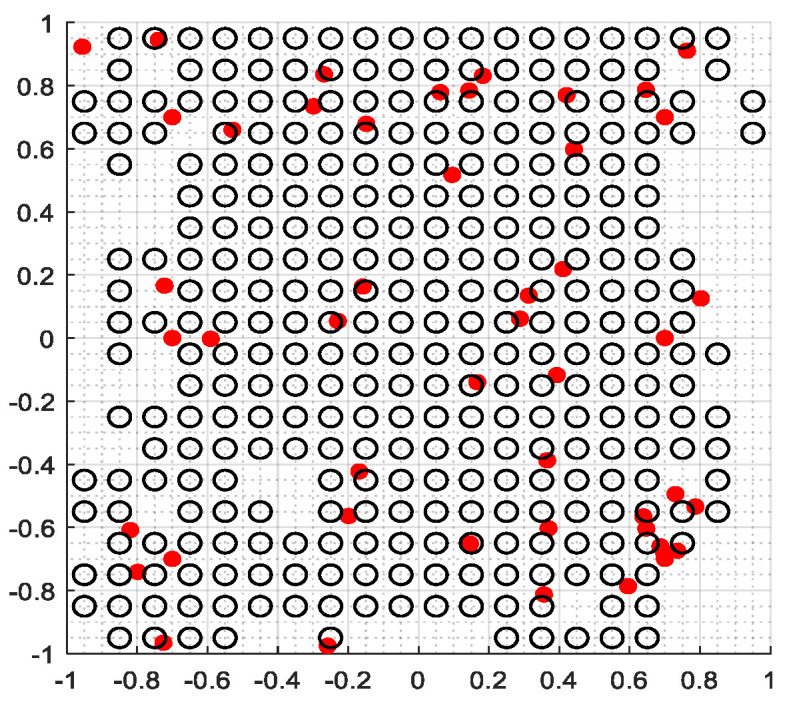
2D viewgraph of optimized deployment for a simple saddle-shaped surface.

**Figure 14:**
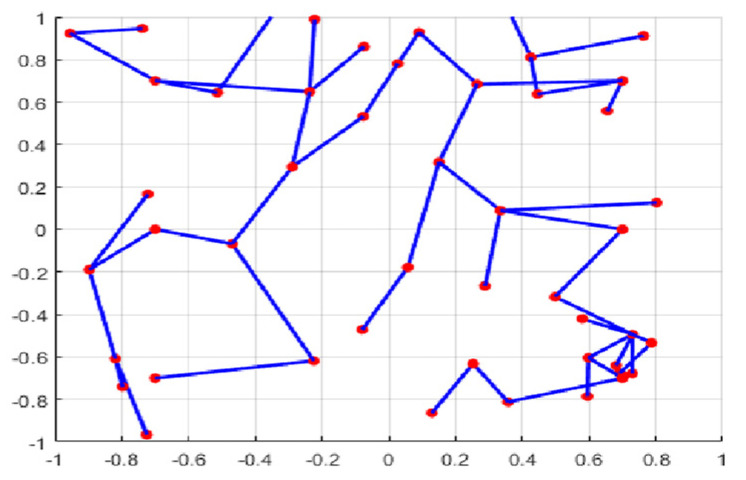
Network connectivity diagram for a simple saddle-shaped surface.

**Figure 15:**
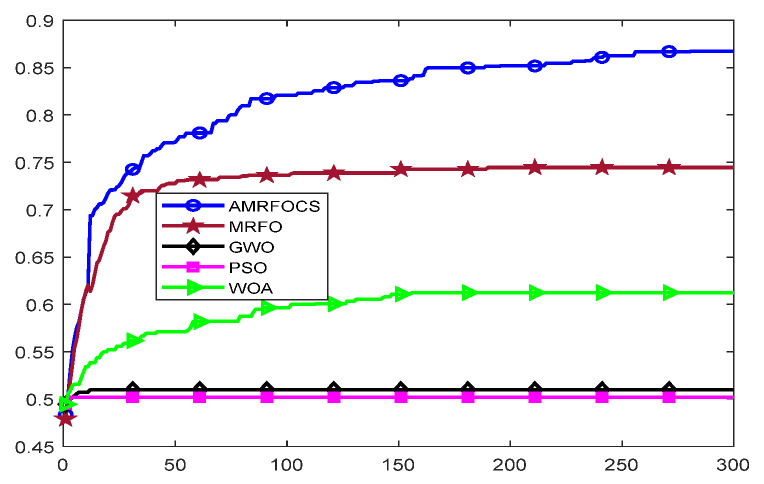
Coverage optimization iterative curve for a simple saddle-shaped surface.

Figure 10, Figure 11, Figure 12, Figure 13 and Figure 14 show the effect of the network coverage optimization of 50 sensor nodes under a simple surface. Figure 10 and Figure 11 show the effect of the initial random deployment of nodes and the optimized node deployment effect. The red dot is the position of the sensor. Initially, the uneven distribution of nodes can be seen from the figure. The optimized node deployment is more uniform, and the coverage area is larger. Figure 12 and Figure 13 are two-dimensional displays of the node distribution and coverage area. In the two-dimensional grid, the black hollow circle means the coverage area of the entire network deployment. The comparison between the initialization of Figure 12 and the optimized coverage area of Figure 13 shows that the effect of the optimized node distribution effect is significant, and the indicators are significantly enhanced. Figure 14 is the minimum spanning tree generated by the Kruskal algorithm mentioned in the network connectivity. Figure 15 is the line chart comparing the coverage of the WSN under network connectivity between the AMRFOCS algorithm and the four comparison algorithms. From the chart, it can be seen that the average coverage of the AMRFOCS optimization is 86.34%; the average coverage of MRFO is 72.63%; and the accuracy is improved by 14%, which is nearly 30% higher than the worst PSO. AMRFOCS has the optimal solution and the highest coverage. The convergence speed of AMRFOCS is also higher than that of the four comparison algorithms.

In order to compare the performance of the algorithm in terms of the network connectivity, Figure 14 and experimental data are combined. With 20 experimental tests and the same number of nodes, AMRFOCS has a higher connectivity and average coverage rate under network connectivity than the other deployment algorithms. AMRFOCS maintains the network connectivity of the WSN under different surfaces and nodes all along the network.

**Figure 16:**
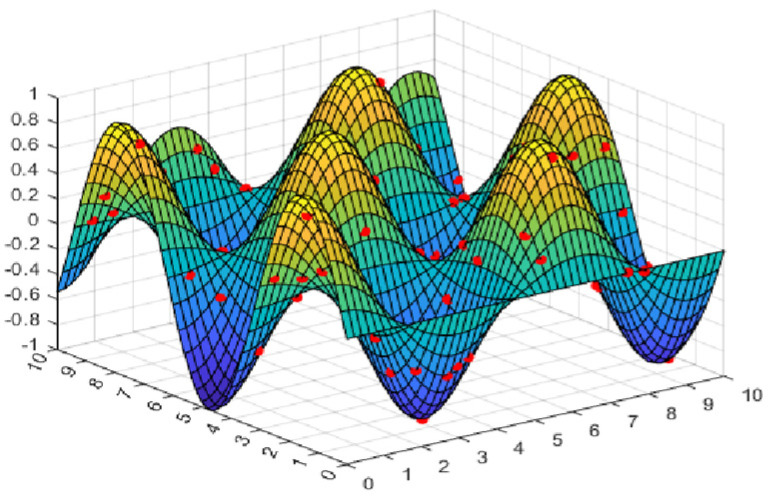
Initial nodes deployment diagram for a complex surface.

**Figure 17:**
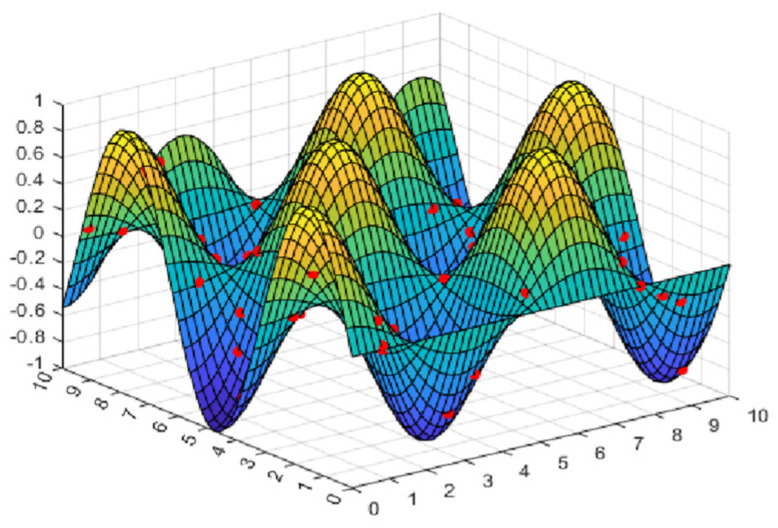
Optimized nodes deployment diagram for a complex surface.

**Figure 18:**
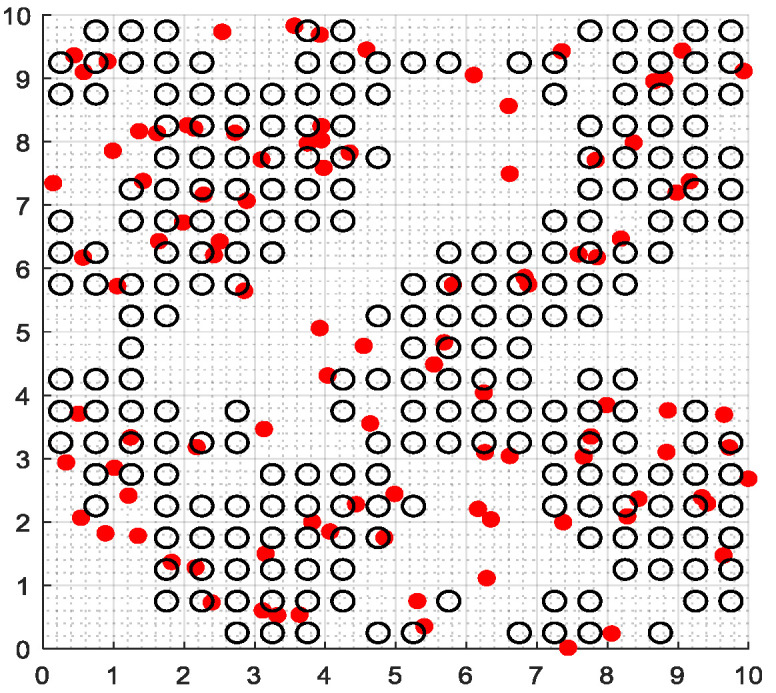
2D viewgraph of initial deployment for a complex surface.

**Figure 19:**
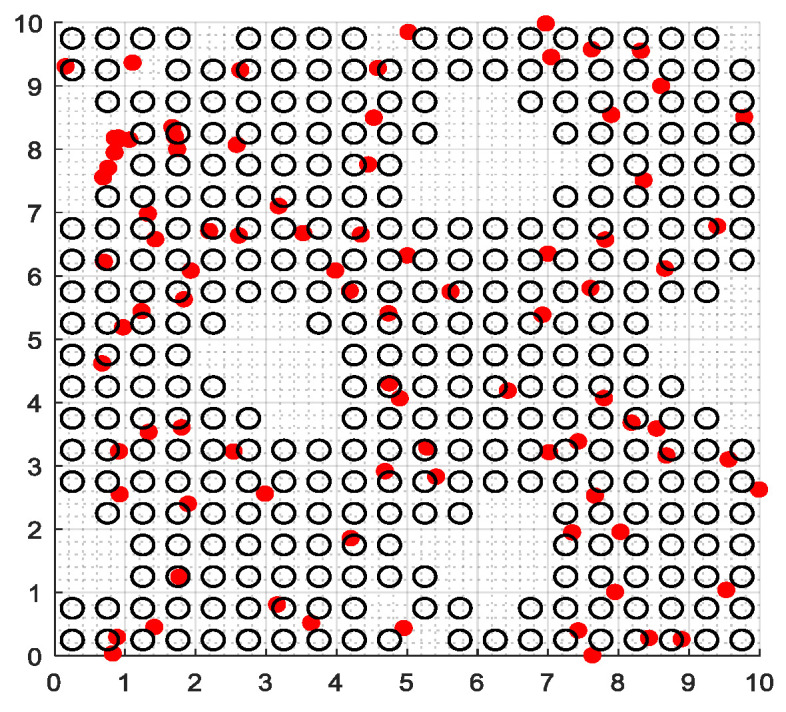
2D viewgraph of optimized deployment for a complex surface.

**Figure 20:**
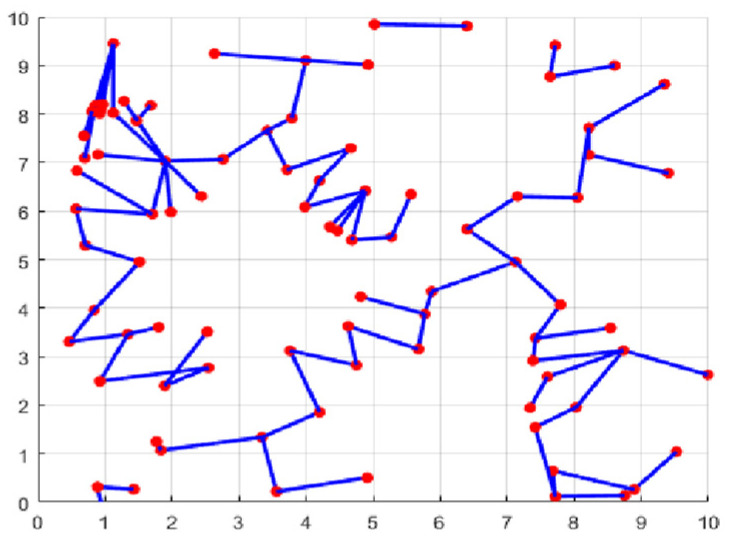
Network connectivity diagram for a complex surface.

**Figure 21:**
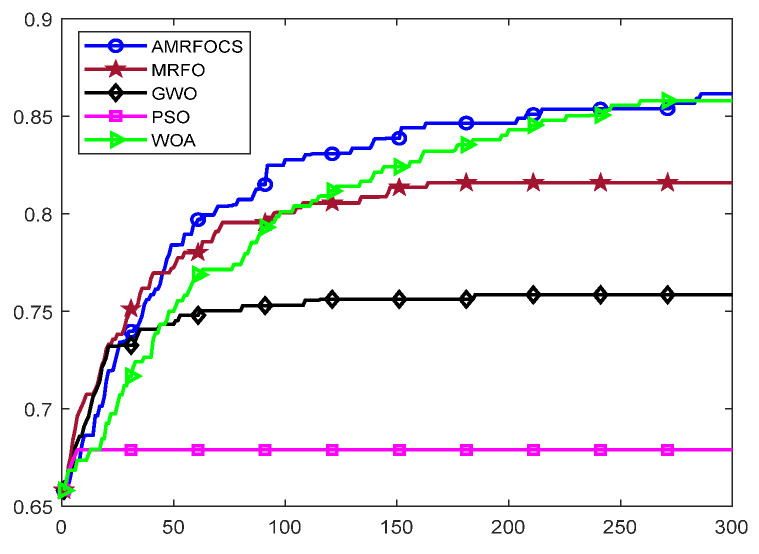
Coverage optimization iterative curve for a complex surface.

Figure 16, Figure 17, Figure 18, Figure 19 and Figure 20 describe the effect of the network coverage optimization of 100 sensor nodes under complex surfaces. Figure 16 and Figure 17 show the effect of the initial random deployment of the nodes and the deployment effect of the nodes optimized by AMRFOCS, respectively. Figure 18 and Figure 19 are two-dimensional displays of the node distribution and coverage area. In the two-dimensional grid, the black circle covers the size of the network deployment. The obvious comparison shows that the initial node distribution is uneven, the optimized node deployment is more uniform and the coverage area is wider. Figure 20 shows the connectivity of the entire network. Figure 21 is the line chart comparing the coverage of the WSN under network connectivity between the AMRFOCS algorithm and the four comparison algorithms including the original MRFO algorithm. The average coverage rate of the AMRFOCS optimization is 85.20%, while MRFO is 81.59%, the accuracy is improved by 4% and it is 17% higher than the worst-performing PSO. AMRFOCS maintains the optimal value and the maximum accuracy.

The similar images in Figure 15 and Figure 21 show the coverage comparison under network connectivity, while the difference is that the overall performance of connectivity and coverage in the deployment algorithm experiment decreased. The main reason is that the complex surface leads to the decrease in the optimization performance of the algorithm and quickly falls into the local optimal state. Comprehensive experiments show that AMRFOCS has good adaptability and it maintains high-quality deployment effects under simple or complex terrain conditions all along the network.

## 6. Conclusions and Future Work

In this paper, an improved Manta Ray Foraging Optimization algorithm (AMRFOCS) hybridized with a Cuckoo Search algorithm is proposed and applied to deploy sensor networks on 3D surfaces. The spiral foraging operator is optimized by combining the random walk of the cuckoo. On this basis, the ability of AMRFOCS to avoid a local optimum and achieve global optimization is effectively improved. In addition, a dynamic disturbance factor is introduced, which can change with the operating state. This strategy helps to coordinate the global search and balance the mining and exploration capabilities of the algorithm. The proposed algorithm is tested on CEC2017 and CEC2020 benchmark datasets. It is compared and tested by using different dimensions and other celebrated and newly developed algorithms. The results manifest that the enhanced algorithm has a superior convergence rate and solution precision. At the same time, through a nonparametric statistical analysis and Wilcoxon signed-rank test, AMRFOCS was found to have good stability and superiority in dealing with a series of experiments.

Simultaneously, AMRFOCS is applied to the network deployment of three-dimensional surfaces. Firstly, the surface area is determined by integration and mesh segmentation, and then the judgment method of the perceptual blind area is improved, so as to achieve the expected experimental results. The experimental results show that AMRFOCS improves the coverage of wireless sensor networks and ensures network connectivity during deployment.

In the next work, the practicality of AMRFOCS deployment on more complex surfaces needs to be further improved, and it can be extended to different application fields, such as image segmentation, feature selection, machine learning, electrical applications and other engineering fields.

## Data Availability

Not applicable.
